# The Role of Beef for the Lowest Cost and Adequate Provision of Bioavailable Nutrients in Modeled Diets at a Population Level in the United States

**DOI:** 10.1016/j.cdnut.2025.107604

**Published:** 2025-11-26

**Authors:** Sylvia MS Chungchunlam, Paul J Moughan

**Affiliations:** Riddet Institute, Massey University, Palmerston North, New Zealand

**Keywords:** diet optimization model, diet cost, nutrient adequacy, dietary requirement, nutrient bioavailability, beef, population, vitamin, mineral

## Abstract

**Background:**

The assumptions that animal-sourced food production is environmentally unsustainable and animal-sourced foods can be seamlessly replaced as a nutrient source by plant-based foods, seldom consider the higher quantity and greater bioavailability of essential nutrients that are naturally present in animal-sourced foods.

**Objectives:**

With beef being a natural food source rich in higher quality protein and bioavailable vitamins, iron, and zinc, the inclusion levels of beef in dietary patterns that meet nutrient requirements at the lowest cost were determined.

**Methods:**

Dietary optimization models using linear programming (LP) were developed to formulate cost-minimized nutrient adequate diets at a population level in the United States. The LP diet models used food compositional data from the USDA, published bioavailability estimates for protein, vitamins, iron, and zinc applied to their contents in foods, daily energy and nutrient requirements for different United States population groups, and food prices from the Thrifty Food Plan 2021 Supplementary datafiles.

**Results:**

Lowest-cost nutrient adequate dietary formulations (total nutrient diets) included both animal-sourced foods (beef liver, milk, eggs, and fish) and plant-based foods, at a daily diet cost ranging from United States $0.73 to United States $1.23. When nutrient contents in foods were given in bioavailable units, rather than total dietary amounts, daily diet cost was higher, ranging from United States $1.75 to United States $7.80, and more animal-derived foods (beef meat, beef liver, milk, eggs, sausages, fish, and clams) were included in the modeled lowest-cost diets (bioavailable nutrient diets). Specifically, beef meat in the bioavailable nutrient diets was the lowest cost main contributor to bioavailable protein, bioavailable vitamin B-12, calcium, phosphorus, selenium, and bioavailable zinc.

**Conclusions:**

Animal-sourced foods, particularly beef meat, were favorably included in adequately nutritious mixed diets formulated at the lowest retail dietary cost for the United States population.

## Introduction

Sustainable food systems are defined around 4 main inter-related dimensions: environment; society; nutrition; and affordability [1]. Recently, the perceived negative environmental impact of animal-sourced food production has prompted dietary patterns to shift towards reducing and replacing animal-sourced foods with plant-based foods [[2], [3], [4]]. The production of red meat, shellfish, and dairy has been particularly viewed as a major contributor to greenhouse gas emissions and inefficient use of land, water, and energy [[3], [4], [5], [6]]. In the United States, beef is produced with high environmental footprints on a weight basis, but trade-offs using other domains of sustainable healthy diets may be considered by weighing the value of societal acceptance, affordability, and nutrient density and bioavailability [7,8]. Guided by sociocultural and personal preferences and organoleptic properties, beef is widely consumed by many populations globally [9,10]. Beef is a common staple food in Western diets, and the daily mean intake of beef for United States children and adolescents aged 2 to 18 y, adults aged 19 to 59 y, and older adults aged 60 and over y, was 31.9 g (1.1 oz), 47.1 g (1.7 oz), and 40.7 g (1.4 oz), respectively [11,12]. The global demand for beef and other animal-sourced foods is expected to rise with high and changing population growth and increasing incomes [3,7,13,14]. From an economic perspective, animal-sourced foods, such as meat, dairy, and eggs, are generally perceived as more costly per unit weight, but due to their higher nutritional value, animal-sourced foods can be positioned as low-cost sources of protein, vitamin A, B vitamins, calcium, iron, and zinc [6,[13], [14], [15], [16]]. At the intersection of societal, economic, and nutritional sustainability dimensions, our previous dietary modeling studies using the linear programming (LP) approach have found that the lowest-cost dietary patterns relied on animal-sourced foods to meet recommended nutrient requirements of the adult population in the United States, New Zealand, and Indonesia [[17], [18], [19]]. However, more focus is needed on growing children, adolescents, and older adults, who are more vulnerable to increases in food prices and have increased nutritional needs [13].

Foods originating from animals are rich natural sources of protein, essential (indispensable) amino acids, vitamins, and minerals [[20], [21], [22]]. Currently in the United States, animal-sourced foods supply nearly all of the calcium, vitamin B-12, and vitamin D required per day, and ∼60% of daily requirements for iron, zinc, niacin, and vitamin B-6 [23]. For example, 100 g (3.5 oz) of cooked lean beef provides >50% of the daily requirements for protein, selenium, vitamin B-12, and zinc for the general United States adult. Indeed, beef contributes key essential nutrients, often identified as falling short of recommended requirements for different populations in the United States [[23], [24], [25], [26]]. Nutritional adequacy depends on the quantity of nutrients in foods and nutrient bioavailability. In addition to providing greater amounts of nutrients, animal-sourced foods contain highly absorbed nutrients that are available for utilization in metabolic functions. Therefore, animal-based foods are generally of higher quality than plant-based foods [5,[20], [21], [22],[27], [28], [29]]. However, plant-based foods do supply bioavailable essential components, such as dietary fiber, vitamin C, and vitamin E, generally not present naturally in animal-sourced foods [20,28,29]. Recently, the digestible indispensable amino acid score (DIAAS) has been established as the most appropriate measure to estimate protein quality, and the DIAAS value is calculated as the lowest ratio of the absorbed amount of the first-limiting essential amino acid to the amino acid reference intake patterns recommended for a target population [27]. Beef has one of the highest DIAAS values, ranging from 92 to 130, and adequately provides all of the dietary essential amino acids [30]. Furthermore, beef is a major contributor to the bioavailable supply of vitamin A, B vitamins, biotin, heme-iron, and zinc [20,28,29,31]. Consideration of nutritional quality, which has been largely overlooked, in bridging nutritional and socioeconomic aspects of sustainable diets, may present a potential advantage for animal-sourced foods, particularly nutrient-rich beef, for different types of consumers. The complexities of the environmental footprints, animal welfare, and human health effects associated with the production and consumption of beef are beyond the scope of this research study [4,6,22,[32], [33], [34]].

The primary objective of the study was to evaluate the inclusion levels of beef in supplying bioavailable protein and amino acids, vitamins, iron, and zinc, to meet nutrient requirements at the lowest dietary cost. Building on our previous dietary modeling studies [17,18], this study aimed to use the same LP approach to assess the respective economic and nutritional roles of animal- and plant-sourced foods in the lowest-cost nutrient adequate dietary patterns for different population groups in the United States. The present study also addressed the effect of nutrient bioavailability, which varies greatly among animal and plant food sources, on the minimum cost and composition of dietary patterns. The hypothesis tested was that animal-sourced foods, particularly beef, would be included in the lowest-cost nutrient adequate dietary patterns population-wide in the United States, due to their cost-effectiveness and high nutrient density and bioavailability.

## Methods

### Dietary optimization models using LP

LP is a commonly applied robust and sensitive mathematical tool used to solve complex dietary mixture problems, and was used in our previous studies [17,18] and here to determine the minimum cost of a nutritionally adequate dietary solution. LP can simultaneously take into account food compositional data, food serving sizes, food prices, and energy and nutrient requirements, to model unique solutions for mixtures of foods, at the lowest price possible. The LP diet optimization models were performed using the Julia language and the JuMP mathematical optimization library, with inherently generated sensitivity analysis outputs, and were displayed using the Pluto reactive interface tool, as detailed in our previous study [18].

The linear objective function of the LP diet models was to minimize dietary cost by changing a set of decision variables, which were the quantities and corresponding costs of selected foods while subjected to several linear restrictions. The linear constraints applied to the LP diet models were limits on food serving sizes (constraint 1), daily estimated energy requirement (constraint 2), acceptable energy contributions from carbohydrate, fat, and protein (constraints 3 and 4), daily minimum (constraint 5), and known maximum (constraint 6) intake requirements for nutrients. The LP cost-minimized diet models can be generalized as follows:

Minimize the cost function

$$f\left(x\right)=\sum_{i=1}^{N_{f}}c_{i}x_{i}$$

Subject to the following constraints:

(1) $$\begin{array}{c} 0\leq x_{i}\leq 2s_{i}\;\left(i=1,2,\ldots ,N_{f}\right) \end{array}$$

(2) $$\begin{array}{c} \sum_{i=1}^{N_{f}}e_{i}x_{i}=E \end{array}$$

(3) $$\begin{array}{c} \left(\;\sum_{i=1}^{N_{f}}e_{ti}x_{i\;}\right)\;/\;E\geq p_{\min} \end{array}$$

(4) $$\begin{array}{c} \left(\;\sum_{i=1}^{N_{f}}e_{ti}x_{i\;}\right)\;/\;E\leq p_{\max} \end{array}$$

(5) $$\begin{array}{c} \sum_{i=1}^{N_{f}}n_{ui}x_{i}\geq \min_{u}\;\left(u=1,2,\ldots ,N_{n}\right) \end{array}$$

(6) $$\begin{array}{c} \sum_{i=1}^{N_{f}}n_{ui}x_{i}\leq \max_{u}\;\left(u=1,2,\ldots ,N_{n}\right) \end{array}$$

where f(x) is the diet cost, $N_{f}$ is the number of foods included in the LP analysis, $c_{i}$ is the cost per unit quantity of food *i*, $x_{i}$ is the unit quantity of food *i*, $s_{i}$ is the daily serving size for food *i*, $e_{i}$ is the energy value per unit quantity of food *i,* E is the daily estimated energy requirement to meet, $e_{ti}$ is the total energy provided by macronutrient (carbohydrate, fat, and protein) *t* per unit quantity of food *i*, $p_{\min}$ is the lower acceptable limit for the proportion of energy provided by macronutrient *t*, $p_{\max}$ is the upper acceptable limit for the proportion of energy provided by macronutrient *t*, $n_{ui}$ is the amount of nutrient *u* per unit quantity of food *i,* $\min_{u}$ is the daily minimum required intake level of nutrient *u,* $N_{n}$ is the number of nutrients included in the LP analysis, and $\max_{u}$ is the daily tolerable highest intake level of nutrient *u*.

Diets formulated using the LP diet model to optimize both cost and nutrient adequacy were theoretical dietary patterns that may not necessarily be practical for consumption. The modeled diets were illustrative of proposed food combinations that comply with daily nutritional recommendations at the lowest cost. Dietary scenarios included: “total nutrient diets,” when nutrient contents in foods were given as total gross dietary amounts, and “bioavailable nutrient diets,” when dietary nutrient contents were given as bioavailable amounts.

### Foods data used in the LP diet models

The most up-to-date and comprehensive list of foods available was sourced from the USDA National Nutrient Database for Standard Reference (SR), Legacy (2018), and supplemented by data from SR, Release 28 (2016) [35,36]. Foods were mainly selected based on the foods that are rich in selected nutrients of public health importance for the total United States population, as described previously [17]. Most mixed dishes, ready meals, snack products, and beverages were not included. The energy and nutrient contents per 100 g of edible portion were based on raw and prepared foods, and appropriate food formats (e.g. canned, frozen, and dried). The list of selected foods ($N_{f}$ = 1977) categorized into 27 main food groups is given in Supplemental Table 1.

Food prices were given in United States $ per 100 g of each food, and pertain to national mean food prices in the United States market for 2021. The estimated June 2021 food prices were obtained from USDA Center for Nutrition Promotion and Policy, mapped to the current Thrifty Food Plan (TFP) using the Purchase to Plate Price Tool, after adjustments for food price inflation from the 2015 to 2016 food prices [37,38]. The food prices corresponding to the Food and Nutrient Database for Dietary Studies food codes from the TFP 2021 Supplementary datafiles were linked to the Nutrient Databank number in the food compositional SR databases, based on food descriptions and formats [35,36,38].

Food serving sizes were expressed as food weights in g per 100 g of food, based on the reference amounts customarily consumed per eating occasion, as were also used in our previous modeling study [17]. To ensure dietary diversity and avoid the inclusion of multiple servings of similar food items belonging to the same food group, serving sizes of food items and food groups were pragmatically constrained to a maximum of 2 serving quantities in the LP diet models (constraint 1).

### Second foods database using nutrient bioavailability estimates in foods

Another foods database was constructed to factor in nutrient bioavailability and present the food contents of protein, folate, niacin, pantothenic acid, riboflavin, thiamin, vitamin A, vitamin B-6, vitamin B-12, vitamin C, vitamin D, vitamin E, vitamin K, iron, and zinc, as bioavailable amounts. The bioavailability of dietary protein is best quantified by the level of crude protein, corrected for digestibility measured at the end of the small intestine and adjusted for endogenous losses [27]. The mean true (standardized) ileal protein digestibility factors were primarily collected from ileostomy studies conducted in adult humans and in the growing pig as an animal model for adult humans, using data from Food and Agriculture Organization (FAO) [27,39] and in-house datasets from the Riddet Institute, Massey University, New Zealand (Table 1). The bioavailability factors for key vitamins in foods were sourced from a previous review study by our group [28], whereby the proportion of the amounts and forms of 13 essential vitamins that are absorbed from different human foods originating from animals and plants and utilized for physiological functions were estimated. The bioavailability factors for iron and zinc were based on estimates of published absorption efficiencies, dependent on the amount of heme-iron and phytate in foods, respectively [20,29,31,40] (Table 1).

**TABLE 1:** True ileal digestibility factors (%) for crude protein applied to food groups, and estimated bioavailability factors (%) for vitamins, iron, and zinc, applied to food sources in the linear programming models

Nutrient	Animal foods		Plant foods		Mixed soups
Protein	Animal proteins	—	Plant proteins	—	86.6
—	Beef	98.1	Legumes	80.9	—
—	Milk	92.8	Plant-based milk and dairy substitutes	84.9	—
—	Dairy products	94.6	Nuts	79.4	—
—	Pork	97.9	Seeds	62.8	—
—	Lamb, Venison	95.2	Vegetables	62.6	—
—	Sausages, cold cuts, and cured meats	98.4	Fruits	52.7	—
—	Chicken	99.0	Breakfast cereal	88.0	—
—	Eggs	92.1	Cereal grains	83.4	—
—	Turkey	98.5	Grains	75.6	—
—	Fish, seafood	94.2	Rice	92.5	—
—	—	—	Pasta and noodles	85.2	—
—	—	—	Sweets, sauces and dressings, fats and oils	77.1	—
Folate	—	66.6	—	54.5	60.6
Niacin	—	67.0	—	57.3	62.2
Pantothenic acid	—	76.3	—	52.0	64.2
Riboflavin	—	61.0	—	64.5	62.8
Thiamin	—	82.3	—	82.7	82.5
Vitamin A	—	74.0	—	15.6	44.8
Vitamin B-6	—	82.8	—	67.7	75.3
Vitamin B-12	—	64.9	—	64.9	64.9
Vitamin C	—	76.4	—	76.4	76.4
Vitamin D	—	80	—	80	80
Vitamin E	—	75	—	75	75
Vitamin K	—	80	—	80	80
Iron	—	19.5	—	7.4	13.5
Zinc	—	30.0	—	15.0	22.5

Data for protein were sourced from FAO [39] and Riddet Institute in-house datasets, data for vitamins were sourced from Chungchunlam and Moughan [28], and data for iron and zinc were sourced from Beal and Ortenzi [20], WHO and FAO [29], Layrisse et al. [31], and Deptford et al. [40].

### Energy and nutrient intake requirements for United States populations

Ten specific age- and sex-grouped populations were studied and comprised: children aged 1 to 3 y; children aged 4 to 8 y; male children aged 9 to 13 y; male adolescents aged 14 to 18 y; male adults aged 19 to 50 y; male adults aged >50 y; female children aged 9 to 13 y; female adolescents aged 14 to 18 y; female adults aged 19 to 50 y; and female adults aged >50 y. The daily estimated energy requirements (EER) for the 10 study populations in the United States are presented in Table 2 [13,41,42], and were exactly fulfilled by the total energy content of modeled LP diets (constraint 2). The constraints for the proportion of energy provided by carbohydrate, fat, and protein, were linearized and incorporated in the LP diet models (constraints 3 and 4). The energy contents derived from dietary carbohydrate, fat, and protein, were calculated based on 4 kcal (16.7 kJ) per g, 9 kcal (37.7 kJ) per g, and 4 kcal (16.7 kJ) per g, respectively. A lower and upper limit was imposed for energy contributed by carbohydrate, fat, and protein, based on acceptable macronutrient distribution ranges (AMDR) for different United States population groups [[41], [42], [43]] (Supplemental Table 2). The AMDR was utilized as a benchmark to construct representative modeled diets that provided these macronutrients proportionally in acceptable broad ranges of total energy at a population level [43].

**TABLE 2:** Cost of modeled lowest-cost nutrient adequate diets, when nutrient contents in foods were given as total dietary amounts (total nutrient diets) or bioavailable amounts (bioavailable nutrient diets), for different population groups in the United States

Population group	Energy value of diet (kcal)	Total nutrient diets		Bioavailable nutrient diets	
		Cost of diet per day (United States $)	Daily cost per 1000 kcal of diet (United States $/1000 kcal)	Cost of diet per day (United States $)	Daily cost per 1000 kcal of diet (United States $/1000 kcal)
Children aged 1–3 y	1000	0.74	0.74	4.19	4.19
Children aged 4–8 y	1500	0.75	0.50	5.56	3.70
Male children aged 9–13 y	2000	1.23	0.61	3.11	1.55
Male adolescents aged 14–18 y	2700	1.11	0.41	4.21	1.56
Male adults aged 19–50 y	2600	1.12	0.43	2.99	1.15
Male adults aged >50 y	2300	1.09	0.48	3.15	1.37
Female children aged 9–13 y	1800	0.73	0.41	3.28	1.82
Female adolescents aged 14–18 y	2100	0.84	0.40	7.80	3.72
Female adults aged 19–50 y	2100	0.84	0.40	No feasible solution^1^	
Female adults aged >50 y	1800	0.87	0.48	1.75	0.97

1 It was not feasible to have bioavailable nutrient diets for female adults aged 19 to 50 y, when nutrient contents in foods were given as bioavailable amounts.

The minimum recommended daily dietary reference intakes of nutrients for 10 different United States population groups were given as either recommended dietary allowances (RDA), to meet the nutritional requirements of 97.5% of all individuals in a population group, or adequate intake (AI), based on determined estimates of the amount of nutrient sufficient for physiological functions [41]. The daily recommended minimum intake requirements of nutrients were met or exceeded by the total amounts of nutrients provided by the modeled LP diets (constraint 5).

For the energy-providing nutrients, AI values were used for linoleic acid (18:2*n*−6), α-linolenic acid (18:3*n*−3), and total dietary fiber, and RDA values were used for protein [41] (Supplemental Table 2). The daily minimum recommended intakes for key vitamins are shown in Supplemental Table 3, and given as RDA values for folate (vitamin B-9), niacin (vitamin B-3), riboflavin (vitamin B-2), thiamin (vitamin B-1), vitamin A, vitamin B-6, vitamin B-12, vitamin C, and vitamin D, and as AI values for choline, pantothenic acid (vitamin B-5), vitamin E, and vitamin K [41,[44], [45], [46], [47], [48]]. As most vitamins occur in foods in different forms, required intake values may be specific to particular forms for some vitamins, such as α-tocopherol for vitamin E, and phylloquinone for vitamin K [41,45,46]. In addition, in the absence of adequate sunlight exposure, daily dietary recommendations for vitamin D were previously reported as AI values for the main form of vitamin D, cholecalciferol, but current recommendations use RDA values for both forms of vitamin D, cholecalciferol (vitamin D-3) and ergocalciferol (vitamin D-2) [41,44,47]. The equivalent contents of some vitamins in foods were also considered. Folate values were expressed as dietary folate equivalents (DFE), whereby 1 unit of DFE is equivalent to 1 unit of food folate, or 0.6 units of folic acid obtained from fortified foods or as a dietary supplement consumed with food [41,48]. Niacin values were expressed as niacin equivalents (NE), whereby 60 units of the essential amino acid tryptophan can be converted to 1 unit of niacin [41,48]. Vitamin A values were expressed in terms of retinol activity equivalent (RAE), whereby 1 unit of RAE is equivalent to 1 unit of retinol (pre-formed vitamin A), 12 units of β-carotene, or 24 units of other pro-vitamin A carotenoids (α-carotene and β-cryptoxanthin) [41,46]. The daily minimum intake requirements for key minerals are shown in Supplemental Table 4, and presented as RDA values for calcium, copper, iron, magnesium, phosphorus, selenium, and zinc, and as AI values for manganese, potassium, and sodium [41,[44], [45], [46], [47],^49^]. Although calcium intake requirements were previously given as estimates of calcium intakes assumed to be adequate (AI), recently RDA values for calcium have been established [41,44,47]. Because daily recommended intakes for potassium and sodium from previous reports are rarely met in practice, the daily AI values for potassium and sodium are presently recommended as lower amounts [41,49,50]. Nutrients that were supplied at exactly 100% of daily minimum intake requirements were referred to as first-limiting.

Although intakes below the minimum recommended adequate levels may lead to deficiencies, excess intakes may result in deleterious health effects. The highest amount of daily nutrient intake that is unlikely to cause adverse health effects to almost all individuals in the population is represented by the tolerable upper intake level (UL) [41]. The total amounts of nutrients provided by the LP modeled diets were limited to the daily recommended maximum intake requirements of nutrients (constraint 6). The UL values used for vitamins and minerals, for different United States population groups are presented in Supplemental Tables 5 and 6, respectively. Values for the UL were available for choline, folate, niacin, vitamin A, vitamin B-6, vitamin C, vitamin D, vitamin E, calcium, copper, iron, manganese, phosphorus, selenium, sodium, and zinc. The UL values for vitamin A apply to pre-formed vitamin A only, but here vitamin A is expressed as RAE, whereby 1 unit of the pre-formed vitamin A retinol is equivalent to 1 unit of RAE [41,46]. The UL values for folate, niacin, and vitamin E apply to synthetic forms obtained from fortified foods or supplements consumed with fortified foods [41,45,48]. It has been noted that excessive consumption of sodium has been linked to a higher risk of high blood pressure (hypertension), and chronic disease risk reduction intake values were used here as upper limits to sodium intake [41,49,50].

### Energy and nutrient intake requirements for the representative United States adult population

For the LP diet model, the mean values for the daily needs for energy and nutrients for male and female adults aged 19 to 50 y were used to represent the general United States adult population. It was estimated that the EER for representative United States adults was 2350 kcal (9832.4 kJ) per day, based on 2600 kcal (10878.4 kJ) per day for male adults aged 19 to 50 y and 2100 kcal (8786.4 kJ) per day for female adults aged 19 to 50 y. It was recommended that for United States adults aged >18 y, 45% to 65% of total energy of the diet should be provided by carbohydrate, 20% to 35% from fat, and 10% to 35% from protein [41]. The daily recommended minimum intakes for representative United States adults were 14.5 g for linoleic acid, 1.35 g for α-linolenic acid, and 31.5 g for dietary fiber. The recommended minimum intake requirement for protein was estimated as 51 g of protein per day for representative United States adults, based on mean values for male adults weighing 70 kg and female adults weighing 57 kg, with a recommended protein intake of 0.80 g per kg body weight per day. For the representative United States adult population, the daily RDA recommendation for vitamins was set at 400 mg DFE for folate, 15 mg NE for niacin, 1.2 mg for riboflavin, 1.15 mg for thiamin, 800 μg RAE for vitamin A, 1.3 mg for vitamin B-6, 2.4 mg for vitamin B-12, 82.5 mg for vitamin C, and 15 μg for vitamin D, and the daily AI recommendation was estimated as 487.5 mg for choline, 5 mg for pantothenic acid, 15 mg for vitamin E, and 105 μg for vitamin K. For the representative United States adult population, the daily RDA recommendation for minerals was 1000 mg for calcium, 900 μg for copper, 13 mg for iron, 362.5 mg for magnesium, 700 mg for phosphorus, 55 μg for selenium, and 9.5 mg for zinc, and the daily AI recommendation was 2.05 mg for manganese, 3000 mg for potassium, and 1500 mg for sodium. For United States adults aged >18 y, the daily UL recommendation was 3500 mg for choline, 1000 μg DFE for folate, 35 mg NE for niacin, 3000 μg RAE for vitamin A, 100 mg for vitamin B-6, 2000 mg for vitamin C, 100 μg for vitamin D, 1000 mg for vitamin E, 2500 mg for calcium, 10,000 μg for copper, 45 mg for iron, 11 mg for manganese, 4000 mg for phosphorus, 400 μg for selenium, 30 mg for sodium, and 40 mg for zinc.

## Results

### Total nutrient diets: modeling lowest-cost nutrient adequate diets for different United States population groups

**FIGURE 1:**
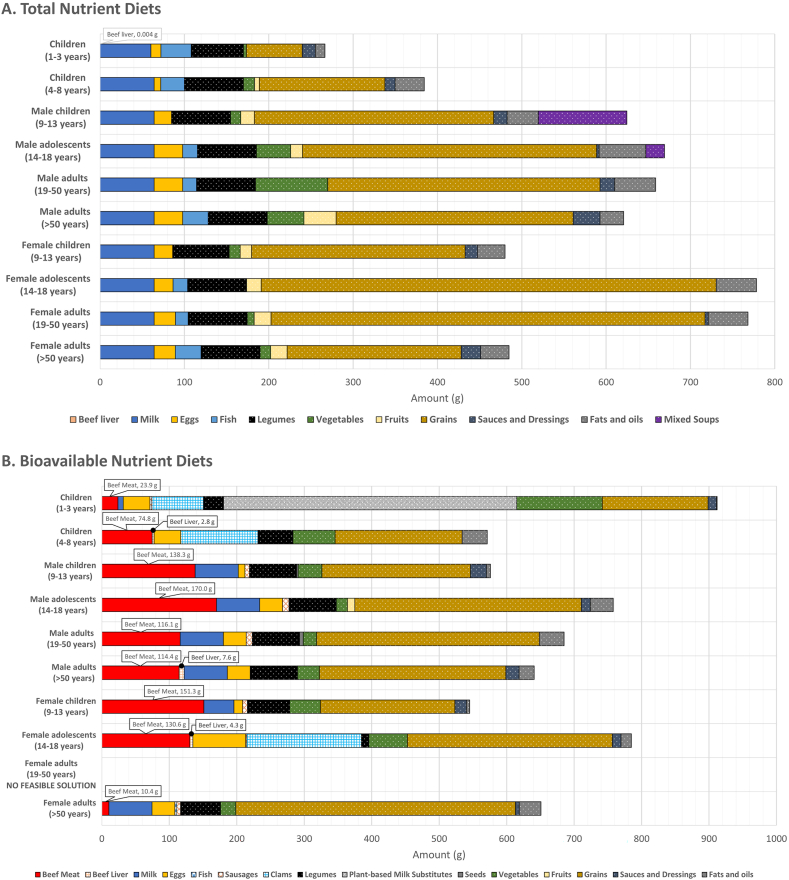
Composition of the modeled lowest-cost nutrient adequate diets for different population groups in the United States, when nutrient contents in foods were given as total dietary amounts ([A] total nutrient diets) or as bioavailable amounts ([B] bioavailable nutrient diets). (B) Bioavailable nutrient diets: for female adults aged 19 to 50 y, a feasible solution was not found when dietary contents of protein, vitamins, iron, and zinc were expressed on a bioavailable basis.

The composition of the modeled lowest-cost mixtures of foods (total nutrient diets), when nutrient contents were given as total gross dietary amounts, that met energy and nutrient needs for different United States population groups is illustrated in Figure 1A. The total nutrient diets included animal-sourced foods, namely milk, eggs, and fish, and beef liver (0.004 g) but only for children aged 1 to 3 y (Figure 1A). The plant-sourced foods included were legumes, vegetables (potatoes, spinach, collard greens, and mushrooms), fruits, grains (oats, wheat-based cereals, corn grits, couscous, pasta, and noodles), sauces and dressings (mayonnaise and soy sauce), fats and oils (vegetable oils and margarine), and mixed green pea soups only for male adolescents aged 14 to 18 y and male adults aged 19 to 50 y (Figure 1A). The cost of the total nutrient diets ranged from United States $0.73/d for female children aged 9 to 13 y to United States $1.23/d for male children aged 9 to 13 y (Table 2). On the same dietary energy basis, the daily cost of the total nutrient diets ranged from United States $0.40 per 1000 kcal for female adolescents aged 14 to 18 y and female adults aged 19 to 50 y to United States $0.74 per 1000 kcal for children aged 1 to 3 y (Table 2).

The first-limiting nutrients provided by the total nutrient diets, at their minimum (100%) intake requirements, for the 10 different United States population groups are shown in Table 3. Supplemental Table 7 shows how the total nutrient diets supplied the required nutrients on a total dietary amount basis. The first-limiting nutrients for all the population groups were choline, vitamin C, vitamin D, vitamin K, and sodium. The essential fatty acid linoleic acid was first-limiting for almost all of the population groups, and close to limiting (106% of requirement) for male adolescents aged 14 to 18 y, whereas the essential fatty acid α-linolenic acid was first-limiting only for all of the children population groups aged 1 to 13 y, male adolescents and adults aged 14 to 50 y, and female adults aged >50 y. Although dietary fiber was first-limiting for all of the children population groups aged 1 to 13 y and all of the male population groups aged >13 y, dietary fiber was supplied in excess of requirement for the all of the female population groups aged >13 y. For children aged 1 to 8 y, vitamin E, calcium and potassium were first-limiting, and vitamin A was met at its minimum required level for children aged 1 to 3 y. For both male children and adolescents aged 9 to 18 y, vitamin E and calcium were first-limiting, and vitamin A was first-limiting for male children aged 9 to 13 y, and nearly at its minimum required level (103%) for male adolescents aged 14 to 18 y, while potassium was also close to being limiting (101-106 % of requirement). For male adults aged >18 y, vitamin E and potassium were first-limiting, and calcium was close to being limiting (103%–107% of requirement). For all of the female population groups aged >8 y, vitamin A and calcium were met at their minimum required levels. While vitamin E and potassium were nearly limiting (101% of requirement) for female children aged 9 to 13 y, potassium was first-limiting for female adults aged >18 y. For United States children aged 1 to 3 y population group, beef liver amounted to 0.004 g and was a low-cost source of the first-limiting vitamin A (0.11% of requirement).

**TABLE 3:** Nutrients provided by the total nutrient diets, when nutrient contents in foods were given as total dietary amounts, were met at their daily minimum intake requirements (first-limiting) for different population groups in the United States

First-limiting nutrients in the total nutrient diets												
Children aged 1–3 years	Linoleic acid	α-linolenic acid	Dietary fiber	Choline	Vitamin A	Vitamin C	Vitamin D	Vitamin E	Vitamin K	Calcium	Potassium	Sodium
Children aged 4–8 y	Linoleic acid	α-linolenic acid	Dietary fiber	Choline	—	Vitamin C	Vitamin D	Vitamin E	Vitamin K	Calcium	Potassium	Sodium
Male children aged 9–13 y	Linoleic acid	α-linolenic acid	Dietary fiber	Choline	Vitamin A	Vitamin C	Vitamin D	Vitamin E	Vitamin K	Calcium	—	Sodium
Male adolescents aged 14–18 y	—	α-linolenic acid	Dietary fiber	Choline	—	Vitamin C	Vitamin D	Vitamin E	Vitamin K	Calcium	—	Sodium
Male adults aged 19–50 y	Linoleic acid	α-linolenic acid	Dietary fiber	Choline	—	Vitamin C	Vitamin D	Vitamin E	Vitamin K	—	Potassium	Sodium
Male adults aged >50 y	Linoleic acid	—	Dietary fiber	Choline	—	Vitamin C	Vitamin D	Vitamin E	Vitamin K	—	Potassium	Sodium
Female children aged 9–13 y	Linoleic acid	α-linolenic acid	Dietary fiber	Choline	Vitamin A	Vitamin C	Vitamin D		Vitamin K	Calcium	—	Sodium
Female adolescents aged 14–18 y	Linoleic acid	—	—	Choline	Vitamin A	Vitamin C	Vitamin D		Vitamin K	Calcium	—	Sodium
Female adults aged 19–50 y	Linoleic acid	—	—	Choline	Vitamin A	Vitamin C	Vitamin D		Vitamin K	Calcium	Potassium	Sodium
Female adults aged >50 y	Linoleic acid	α-linolenic acid	—	Choline	Vitamin A	Vitamin C	Vitamin D		Vitamin K	Calcium	Potassium	Sodium

### Bioavailable nutrient diets: modeling lowest-cost nutrient adequate diets for different United States population groups, when nutrient contents in foods were given as bioavailable amounts

**FIGURE 2:**
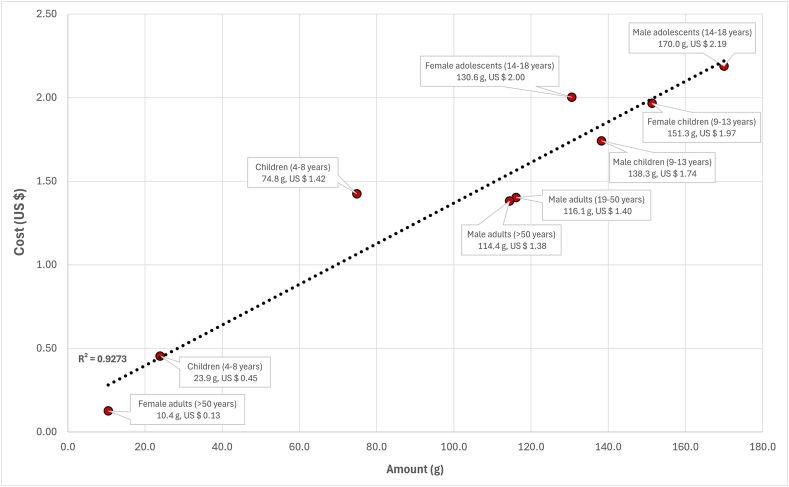
The cost (United States $) and amount (g) of beef meat in the modeled lowest-cost dietary patterns (bioavailable nutrient diets), when nutrient contents in foods were given as bioavailable amounts.

When the contents of protein, folate, niacin, pantothenic acid, riboflavin, thiamin, vitamin A, vitamin B-6, vitamin B-12, vitamin C, vitamin D, vitamin E, vitamin K, iron, and zinc, in foods were expressed on a bioavailable basis, it was not feasible to model lowest-cost dietary solutions that met all of the energy and nutrient requirements for female adults aged 19 to 50 y. Figure 1B shows the composition of the modeled lowest-cost nutrient adequate dietary patterns (bioavailable nutrient diets) for the 9 United States population groups, where feasible solutions were obtained. Beef meat (10.4–151.3 g) was selected for all of the population groups, with the addition of beef liver for children aged 4 to 8 y (2.8 g), female adolescents aged 14 to 18 y (4.3 g), and male adults aged >50 y (7.6 g) (Figure 1B). Bioavailable nutrient diets included beef meat, beef liver, milk, eggs, fish, pork liver sausages, and clams, as foods sourced from animals (Figure 1B). Foods that originated from plants were legumes, plant-based milk substitutes beverages, seeds, vegetables (mushrooms, spinach, cabbage, cauliflower, and yambean), fruits, grains (oats, wheat-based cereals, breakfast ready-to-eat cereals low and high in sugar, corn grits, and bread), sauces and dressings (mayonnaise and soy sauce), and fats and oils (vegetable oils and margarine) (Figure 1B). The daily diet cost of the bioavailable nutrient diets was higher compared with the total nutrient diets, ranging from United States $2.99 for male adults aged 19 to 50 y to United States $7.80 for female adolescents aged 14 to 18 y (Table 2). Beef liver accounted for 0.6%, 0.6%, and 2.7% of total diet cost, relative to the amount of beef liver in the bioavailable nutrient diets for children aged 4 to 8 y (2.8 g, 0.5% of total food weight), female adolescents aged 14 to 18 y (4.3 g, 0.6% of total food weight), and male adults aged >50 y (7.6 g, 1.2% of total food weight), respectively (data not shown). The cost and amount of beef meat in the bioavailable nutrient diets are shown in Figure 2. Beef meat contributed between 7.2% of total diet cost for female adults aged >50 y to 59.9% for female children aged 9 to 13 y, which was proportional to beef meat amounting between 1.6% of total food weight for female adults aged >50 y and 27.8% of total food weight for female children aged 9 to 13 y, respectively (data not shown). The daily cost per 1000 kcal of the bioavailable nutrient diets was lowest at United States $0.97 for female adults aged >50 y and highest at United States $4.19 for children aged 1 to 3 y (Table 2).

When nutrient contents in foods were given as bioavailable amounts in the bioavailable nutrient diets, the first-limiting nutrients that were met at their daily minimum required levels (100%) for all of the 9 feasible United States population groups are shown in Table 4. Supplemental Table 8 shows how the requirements for nutrients were fulfilled by the bioavailable nutrient diets on a dietary bioavailable nutrient basis. For all of the 9 feasible population groups, linoleic acid, α-linolenic acid, bioavailable vitamin A, bioavailable vitamin K, bioavailable iron, and bioavailable zinc were first-limiting. For children aged 1 to 8 y, dietary fiber, choline, calcium, and potassium were also first-limiting, and bioavailable vitamin D was met at its minimum required level for only children aged 1 to 3 y. While dietary fiber, choline, bioavailable vitamin D, and sodium were first-limiting for male children aged 9 to 13 y, bioavailable vitamin C and bioavailable vitamin D were first-limiting for male adolescents aged 14 to 18 y. Similarly, for both male adults population groups, choline, potassium, and sodium were first-limiting, but bioavailable vitamin C and bioavailable vitamin D were supplied at their minimum requirements for male adults aged 19 to 50 y, and close to requirements (102%) for male adults aged >50 y. Other nutrients that became first-limiting for female children aged 9 to 13 y were dietary fiber, choline, bioavailable vitamin D, and sodium. Calcium and potassium were first-limiting for female adolescents aged 14 to 18 y and female adults aged >50 y, but the requirements for dietary fiber were met at minimum for female adolescents aged 14 to 18 y, and the requirements for bioavailable vitamin D were achieved at minimum for female adults aged >50 y.

**TABLE 4:** Nutrients provided by the bioavailable nutrient diets, when nutrient contents in foods were given as bioavailable amounts, were met at their daily minimum intake requirements (first-limiting) for different population groups in the United States^1^

First-limiting nutrients in the bioavailable nutrient diets													
Children aged 1–3 y	Linoleic acid	α-linolenic acid	Dietary fiber	Choline	Vitamin A	—	Vitamin D	Vitamin K	Calcium	Iron	Potassium	—	Zinc
Children aged 4–8 y	Linoleic acid	α-linolenic acid	Dietary fiber	Choline	Vitamin A	—	—	Vitamin K	Calcium	Iron	Potassium	—	Zinc
Male children aged 9–13 y	Linoleic acid	α-linolenic acid	Dietary fiber	Choline	Vitamin A	—	Vitamin D	Vitamin K	—	Iron	—	Sodium	Zinc
Male adolescents aged 14–18 y	Linoleic acid	α-linolenic acid	—	—	Vitamin A	Vitamin C	Vitamin D	Vitamin K	—	Iron	—	—	Zinc
Male adults aged 19–50 y	Linoleic acid	α-linolenic acid	—	Choline	Vitamin A	Vitamin C	Vitamin D	Vitamin K	—	Iron	Potassium	Sodium	Zinc
Male adults aged >50 y	Linoleic acid	α-linolenic acid	—	Choline	Vitamin A	—	—	Vitamin K	—	Iron	Potassium	Sodium	Zinc
Female children aged 9–13 y	Linoleic acid	α-linolenic acid	Dietary fiber	Choline	Vitamin A	—	Vitamin D	Vitamin K	—	Iron	—	Sodium	Zinc
Female adolescents aged 14–18 y	Linoleic acid	α-linolenic acid	Dietary fiber	—	Vitamin A	—	—	Vitamin K	Calcium	Iron	Potassium	—	Zinc
Female adults aged 19–50 y	No feasible solution^2^												
Female adults aged >50 y	Linoleic acid	α-linolenic acid	—	—	Vitamin A	—	Vitamin D	Vitamin K	Calcium	Iron	Potassium	—	Zinc

1 The contents of vitamin A, vitamin C, vitamin D, vitamin K, iron, and zinc, in foods, were given as bioavailable amounts.

2 For female adults aged 19 to 50 y, a feasible solution was not found.

Beef meat and beef liver were important low-cost sources for the first-limiting micronutrients. For United States female adults aged >50 y, a small portion of beef meat (10.4 g, 1.6% of total food weight) supplied calcium (9.1% of requirement), bioavailable zinc (3.6% of requirement), bioavailable iron (0.68% of requirement), bioavailable vitamin K (0.15% of requirement), bioavailable vitamin A (0.09% of requirement), and bioavailable vitamin D (0.05% of requirement). For United States female children aged 9 to 13 y, a bigger serving of beef meat (151.3 g, 27.8% of total food weight) provided bioavailable zinc to 54.3% of requirement, choline to 35.2% of requirement, bioavailable iron to 10.1% of requirement, and <10% of requirement for sodium (8.1%), bioavailable vitamin K (3.2%), bioavailable vitamin A (1.3%), and bioavailable vitamin D (0.81%). For United States children aged 4 to 8 y, beef meat (74.8 g, 13.1% of total food weight) contributed calcium (50.4% of requirement), bioavailable zinc (50.3% of requirement), and choline (28.2% of requirement), and beef liver (2.8 g, 0.5% of total food weight) was the greatest contributor to bioavailable vitamin A (49.0% of requirement). For United States female adolescents aged 14 to 18 y, beef meat (130.6 g, 16.6% of total food weight) provided >80% of the RDA requirement for calcium (83.0%) and >40% of the RDA requirement for bioavailable zinc (44.3%), and beef liver (4.3 g, 0.6% of total food weight) accounted for >40% of the RDA requirement for bioavailable vitamin A (43.2%). For United States male adults aged >50 y, beef meat (114.4 g, 17.9% of total food weight) supplied bioavailable zinc (29.0% of requirement) and choline (17.8% of requirement), and beef liver (7.6 g, 1.2% of total food weight) contributed bioavailable vitamin A (59.3% of requirement).

### Diets for United States adults: modeling lowest-cost nutrient adequate diets for the representative United States adult population group

Given the constraints imposed on the LP diet model and when dietary contents of protein, vitamins, iron, and zinc were expressed on a bioavailable basis, it was not feasible to obtain modeled lowest-cost nutrient adequate dietary patterns (bioavailable nutrient diets) for female adults aged 19 to 50 y, but it was achievable to model for the general United States adult population group, the latter based on the mean energy and nutrient requirements for male and female adults aged 19 to 50 y.

**FIGURE 3:**
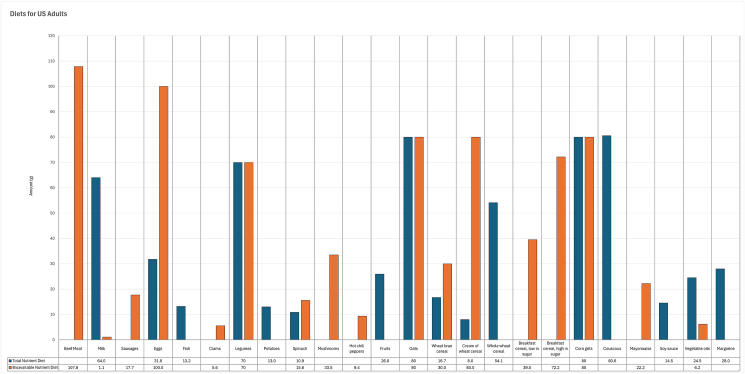
Composition of the modeled lowest-cost nutrient adequate diets for the representative adult population group in the United States (diets for United States adults), when nutrient contents in foods were given as total dietary amounts (total nutrient diet) or as bioavailable amounts (bioavailable nutrient diet).

The composition of modeled lowest-cost diets for United States adults is presented in Figure 3. On the basis of total dietary nutrient contents, the total nutrient diet contained 17.7% of animal-based foods, namely milk (10.4%), eggs (5.2%), and fish (2.1%), and 82.3% of plant-based foods, including legumes, potatoes, spinach, fruits, energy-dense grains, sauces, and fats and oils (Figure 3). On the basis of bioavailable nutrient contents in foods, the composition of the bioavailable nutrient diet was more varied with 30.1% of animal-sourced foods, including beef meat (14.0%), milk (0.1%), pork liver sausages (2.3%), eggs (13.0%), and clams (0.7%), and 69.9% of plant-based foods (Figure 3). The daily diet cost was United States $0.96 (United States $0.41 per 1000 kcal) and United States $4.39 (United States $1.87 per 1000 kcal) for the total nutrient diet and bioavailable nutrient diet, respectively. Animal-sourced foods accounted for 52.0% of total diet cost, notably 36.5% for beef meat in the bioavailable nutrient diet, compared with 27.0% of total diet cost contributed by animal-sourced foods in the total nutrient diet (data not shown). The total nutrient diet provided daily 2350 kcal (9832.4 kJ), 320.8 g of carbohydrate, 78.3 g of fat, and 78.0 g of protein, and the energy proportions derived from carbohydrate (55.8%), fat (30.6%), and protein (13.6%) were within the AMDR values of 45% to 65% for carbohydrate, 20% to 35% for fat, and 10% to 35% for protein, respectively [41]. Similarly, the bioavailable nutrient diet supplied 339.9 g of carbohydrate, 68.7 g of fat, and 93.1 g of protein, with respective energy contributions of 57.9%, 26.3%, and 15.8%.

**FIGURE 4:**
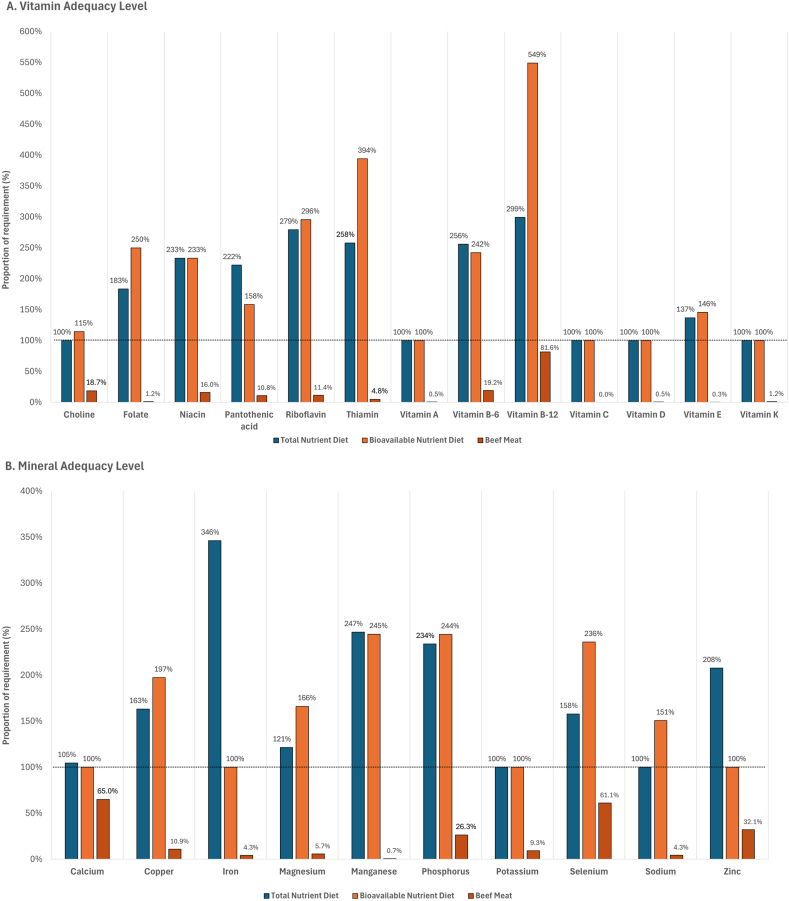
Proportion of required vitamins ([A] vitamin adequacy level) and minerals ([B] mineral adequacy level), provided by the modeled lowest-cost nutrient adequate diets for the representative adult population group in the United States, when nutrient contents in foods were given as total dietary amounts (total nutrient diet) or as bioavailable amounts (bioavailable nutrient diet). The contribution of 100 g (3.5 oz) of beef meat to daily vitamin and mineral requirements is also reported (beef meat).

Regardless of whether protein, vitamins, iron, and zinc were given on a total dietary or bioavailable basis, the nutrients that remained first-limiting for both the total nutrient diet and bioavailable nutrient diet for United States adults were linoleic acid, α-linoleic acid, vitamin A, vitamin C, vitamin D, vitamin K, and potassium. Dietary fiber was found to be first-limiting for the total nutrient diet, but was sufficiently provided (128% of requirement) by the bioavailable nutrient diet, whereas protein was supplied in excess of requirement, with 153% and 183% for the total nutrient diet and bioavailable nutrient diet, respectively. For United States adults, the requirements for vitamins (Figure 4A) and minerals (Figure 4B) were adequately met. Choline and sodium were also found to be first-limiting for the total nutrient diet. When the modeling was done on the basis of bioavailable nutrients, iron and zinc were also first-limiting for the bioavailable nutrient diet.

For United States adults, beef meat (107.8 g, 3.8 oz, 14.0% of total food weight) included in the bioavailable nutrient diet provided the first-limiting calcium to 70.1% of RDA requirement and the first-limiting bioavailable zinc to 34.6% of RDA requirement. A serving of 100 g (3.5 oz) of cooked beef meat contributed protein to 55.0% of daily requirement, vitamin B-12 to 81.6% of requirement, calcium to 65.0% of requirement, selenium to 61.1% of requirement, zinc to 32.1% of requirement, phosphorus to 26.3% of requirement, and also provided other essential vitamins and minerals, as presented in Figure 4.

The shadow prices resulting from the sensitivity of daily minimum cost of modeled diets to an infinitesimal relaxation of nutrient constraints for United States adults are reported in Supplemental Table 9. Daily diet cost was influenced by increasing the constraint on the daily UL intake level for folate, niacin, and iron. The shadow prices indicated that decreasing the daily minimum intake requirements for linoleic acid, α-linolenic acid, choline, vitamin A, vitamin C, vitamin D, vitamin K, calcium, potassium, and sodium would result in a reduction in daily diet cost. Bioavailable iron and bioavailable zinc had the highest shadow prices (–0.994 and –0.648, respectively), to predict a possible reduction in daily diet cost (United States $4.39 for the bioavailable nutrient diet) per unit decrease in the daily recommended intake requirements for iron and zinc.

## Discussion

Nutrient bioavailability is often overlooked in the context of sustainable diets, and needs attention to prioritize affordable nutritious foods and ensure food and nutrient security for the growing global population. Animal-sourced foods are rich in essential nutrients that are often more readily absorbed and bioavailable than those found in plant-based foods [6,13,16,[20], [21], [22],[27], [28], [29], [30], [31]]. One such high-quality animal-based food source is beef meat, which has high contents of bioavailable protein, vitamins, and minerals.

The present study involved the development and methodological application of LP as a mathematical optimization modeling tool to take into account nutrient bioavailability and determine the inclusion levels of beef in nutrient adequate dietary patterns formulated at the lowest cost, population-wide in the United States. The United States has good comprehensive food and cost data [[35], [36], [37], [38]], and was used here as an exemplary economy. Similar to our previous LP studies, animal-sourced foods, such as milk, eggs, and fish, were found to be included in the lowest-cost diet formulations that met energy and nutrient requirements throughout the lifespan of the United States populations, when nutrient contents in foods were given on a total gross dietary (not bioavailable) basis [17,18]. When bioavailability was considered for dietary contents of protein, vitamins, iron, and zinc, a greater variety of animal-sourced foods was selected to be part of modeled lowest-cost diets, including milk, sausages, eggs, fish, beef meat, and beef liver. Our LP diet analyses showed that nutrient bioavailability plays an important role in supporting intake of nutrient-dense animal-sourced foods to help provide bioavailable essential nutrients and meet the nutrient needs of the United States population, particularly for some young and older United States population groups. Moreover, environmentally, Barre et al. [51] found that accounting for protein quality and bioavailability of vitamin A, iron, and zinc, resulted in similar quantities of ruminant meat in modeled nutrient adequate dietary patterns with reduced diet-related environmental impacts, in terms of greenhouse gas emissions, eutrophication, and acidification, for French adults. Furthermore, Moughan [5] demonstrated that consideration of protein quality attenuates the environmental outcomes of the production of animal proteins in comparison with plant proteins, by expressing greenhouse gas emissions, land use, and freshwater use, per kg of true ileal digestible amino acid lysine rather than per 100 g of total protein. Some have argued that the consumption of animal-based foods has associated environmental and health costs [4,22,33,52]. These potential concerns are not addressed here. This study has considered diet nutrient delivery to the body and dietary cost. Our work shows that when bioavailable nutrients in foods are considered, animal-sourced foods are included in the lowest-cost dietary patterns that meet stated nutrient requirements. Further research is needed to correct for protein quality and include bioavailability of micronutrients when evaluating the metrics for the environmental footprints and other externalities related to food production and dietary patterns, which is beyond the scope of the present study.

The focus of this study was to use LP to model lowest-cost diets to meet dietary recommendations for different members of the United States population. Male children aged 9 to 13 y and female adolescents aged 14 to 18 y faced the highest diet costs, and children aged 1 to 3 y had the highest diet cost per 1000 kcal. Using a similar approach globally, Bai et al. [53] found that in an observational study from 172 countries around the world, the global median cost of lowest-cost nutrient adequate diets was highest for children aged 9 to 13 y and adolescents aged 14 to 18 y, thereby emphasizing that growing children and adolescents with their increased nutrient needs are most susceptible to changes in food prices [13]. Considering nutrient bioavailability in the current LP diet models led to greater daily diet costs, ∼50% to 89% more, due to the selection of more animal-sourced foods to supply key essential bioavailable nutrients closer to requirements. Other diet modeling studies indicated that replacing animal-derived foods with plant-based foods resulted in similar increases in diet cost, ∼45% more, and highlighted intakes of selected nutrients as cause for concern for the United States population [17,54]. The essential fatty acids, dietary fiber, choline, most vitamins, and minerals were the first-limiting nutrients driving the cost for both dietary scenarios across the whole United States population. The minimum cost estimates of nutrient adequate diets, from empirical observations or generated by LP models, provide a useful cost metric to monitor the ability of different foods for their provision of essential nutrients [13,17,18,40,53,54]. A diverse range of animal- and plant-sourced foods in the lowest-cost dietary patterns will assist in providing bioavailable nutrients to adequately meet dietary requirements. Plant-based foods were cost-effective sources of the first-limiting dietary fiber, bioavailable vitamin C, and bioavailable vitamin K, whereas animal-sourced foods were cost-effective sources of the first-limiting choline, bioavailable vitamin A, bioavailable iron, and bioavailable zinc. Beef meat was the lowest-cost contributor to bioavailable protein, bioavailable vitamin B-12, and other B-group vitamins, calcium, phosphorus, selenium, and bioavailable zinc. Previous studies have also shown that the consumption of beef is important to provide essential shortfall nutrients and achieve nutritional adequacy for different population groups in the United States [[23], [24], [25], [26],^55^,^56^]. Although the cost of animal-sourced foods per unit weight appears to be relatively high, animal-based foods tend to be on the lower end when food costs are expressed per unit nutrient [6,15,16,57]. In this instance, when food prices are presented in terms of the provision of bioavailable nutrients to meet recommended intakes, the cost of beef per unit bioavailable nutrient strengthens the argument for the cost-effective role of beef in dietary patterns. Moreover, shifting emphasis from cost per unit weight to cost per unit nutrient and cost per unit bioavailable unit provides a broader view of purchasing power to enhance the nutritional value and quality of dietary patterns for consumers. This study aimed to identify the economic feasibility of different foods of high nutritional quality within the lowest cost and nutrient adequate dietary patterns, and may help to guide food-based dietary interventions to improve nutrition at a tangible cost for the United States population.

One important finding of this study was that it was infeasible within the LP model constraints and when nutrient bioavailability was considered, to achieve the lowest-cost nutritionally adequate dietary patterns for female adults aged 19 to 50 y. There is much uncertainty around recommended minimum intake requirement levels, which are generally given as bioavailable amounts, and may be deemed too high [29,41]. Our LP diet model tool was designed to be flexible and allow model users to adjust the energy and nutrient requirements of a selected population group. By relaxing nutrient requirement constraints for the 19 to 50 y aged female adult grouping, in particular for iron and zinc, feasible solutions can be obtained. In addition, there is still very little knowledge and no consensus available on the bioavailability of minerals and trace elements among animal- and plant-based foods, though it is generally agreed that animal-sourced foods have higher contents of highly bioavailable minerals compared with plant-sourced foods. This study used the best available estimates for bioavailable amounts of iron and zinc in foods, as a proxy for mineral bioavailability [20,29,31,40]. The bioavailability of iron and zinc is influenced by host-related factors, such as an individual’s iron or zinc status, and is highly dependent on food source, and the presence of enhancing food components, such as vitamin C that can increase the absorption of iron, or inhibiting food components, such as phytates that can reduce absorption of iron, zinc and other minerals [29,41,[58], [59], [60]]. Nonetheless, in the case of iron and zinc, these differences in food-related bioavailability are considered in setting recommended daily intake values [29,[58], [59], [60], [61], [62]]. The dietary supply of bioavailable iron and bioavailable zinc to meet requirements for United States populations of all ages was identified as influential limiting factors, also indicated by their high shadow prices on daily cost of the lowest-cost dietary solutions feasible for the representative United States adult population. This is in line with several studies that demonstrated the importance of considering the bioavailability of iron and zinc when modeling dietary patterns that met nutrient requirements [40,51,58,63,64]. The findings presented here imply that the bioavailability of iron and zinc was the main player driving diet feasibility and cost, and that nutrient bioavailability is a critical factor that can impact the utilization of foods, such as beef meat, in low-cost nutritious dietary formulations.

It is important to note that our results need to be interpreted with some caution, and should not be extended beyond the realm of the current LP model and the associated data and constraints. Although snack products and beverages were excluded as not being nutritionally relevant, food items included in the list of foods in the foods database were considered to be appropriate to be used to make mixed dishes and ready meals. The range of linear constraints applied to the LP diet models has also been clearly articulated in this study. This current study has some specific limitations. The inclusion of fortified foods in the foods datasets may have been responsible for their selection in modeled lowest-cost diets, particularly cereal grains and breakfast ready-to-eat cereals fortified with vitamins, iron, and other minerals [17,18]. Fortification of energy-dense foods that naturally contain these nutrients in minimal quantities provides an inexpensive source of these essential nutrients [65]. Food prices pertained to mean national retail food prices for the year 2021 in the United States market [37,38], and do not consider regional differences, seasonal local availability, market price fluctuations, food price subsidies, and national retail food price policies. To explore food price elasticities to some degree, our LP diet model exercise does feature user interfaces to change the overall food costs of particular food groups. Diet affordability by weighing diet cost in relation to prevailing incomes and food expenditure shares was also not measured in this study. However, other diet optimization studies are available to formulate diets at minimum cost to obtain adequate daily nutrition, and compare with the food purchasing economic ability. The cost of the diet method and software was created using LP to address nutritional challenges facing individuals and households at the financial poverty line [40]. A similar approach using non-LP was applied to develop the TFP for the lowest-cost diet that meets dietary recommendations for different populations in the United States, focused on food-secure American households [37]. In the absence of data regarding the sociocultural acceptability, holistic properties, and organoleptic quality of foods, the proposed dietary composition of the modeled diets is theoretical and does not imply practical, realistic diets for consumption. The approach, however, does realistically identify meaningful foods that provide essential nutrients at a relatively low price, guiding dietary choices aligned with low-cost nutrient adequate dietary patterns and nutrient sensitivity.

In conclusion, when the LP approach was used to formulate the lowest cost nutritionally balanced diets, and when nutrient bioavailability was factored in, animal-sourced foods, particularly beef meat, were included in modeled mixed diets. This demonstrates that, for the prevailing conditions, animal-based foods are cost effective and favorably included for the adequate provision of key essential nutrients. Specifically, beef meat is an economically viable food source of bioavailable protein, bioavailable B-group vitamins, bioavailable iron, and bioavailable zinc, essential nutrients that are frequently in shortfall in human diets. Consideration of bioavailability has implications for highlighting value-added foods in sustainable diets, leveraging nutrient intake and dietary recommendations, and ensuring nutrient adequacy and food security for different populations globally.

## Author contributions

The authors’ responsibilities were as follows – SMSC: devised the model and performed model simulations, wrote the paper with input from PJM, and had primary responsibility for final content; and all authors: designed the research, conducted the research, analyzed the data, and read and approved the final manuscript.

## Data availability

The data and analytic codes described in the manuscript will be made available upon request to the corresponding author.

## Funding

The research was funded by the Beef Checkoff. The funders had no role in study design, collection, analysis, and interpretation of data, writing of the report, and decision to publish.

## Conflict of interest

The authors report no conflicts of interest.
