# Corrigendum to “The Role of Beef for the Lowest Cost and Adequate Provision of Bioavailable Nutrients in Modeled Diets at a Population Level in the United States” Current Developments in Nutrition, Volume 9, Issue 12, 107604, Dec 2025

**DOI:** 10.1016/j.cdnut.2026.107666

**Published:** 2026-03-10

**Authors:** Sylvia MS Chungchunlam, Paul J Moughan

**Affiliations:** Riddet Institute, Massey University, Palmerston North, New Zealand

The authors regret that several errors were noted in the original publication. This corrigendum provides the rationale for the re-analysis of the data, includes revised Tables and Figures, and corrections as listed below.

1. In the Abstract section, the following sentences in the Results have been revised to: “Lowest-cost nutrient adequate dietary formulations (total nutrient diets) included both animal-sourced foods (beef liver, milk, eggs, and fish) and plant-based foods, at a daily diet cost ranging from United States $0.73 to United States $1.12. When nutrient contents in foods were given in bioavailable units, rather than total dietary amounts, daily diet cost was higher, ranging from United States $1.66 to United States $7.59, and more animal-derived foods (beef meat, beef liver, milk, eggs, sausages, fish, clams, and chicken) were included in the modeled lowest-cost diets (bioavailable nutrient diets). Specifically, beef meat in the bioavailable nutrient diets was the lowest cost main contributor to bioavailable protein, bioavailable vitamin B-12, phosphorus, selenium, and bioavailable zinc.”
2. In the Results section, for Total nutrient diets: modeling lowest-cost nutrient adequate diets for different United States population groups, there were incorrect values found for the cost of the total nutrient diets. For male children aged 9 to 13 y, the corrected daily cost of the total nutrient diets has been revised to United States $0.81 and United States $0.40 per 1000 kcal. Table 2 has been updated as appended below.

Based on this change, the following sentences in the Results section have been corrected to: “The cost of the total nutrient diets ranged from United States $0.73/d for female children aged 9 to 13 y to United States $1.12/d for male adults aged 19 to 50 y (Table 2). On the same dietary energy basis, the daily cost of the total nutrient diets ranged from United States $0.40 per 1000 kcal for male children aged 9 to 13 y, female adolescents aged 14 to 18 y, and female adults aged 19 to 50 y to United States $0.74 per 1000 kcal for children aged 1 to 3 y (Table 2).”

**TABLE 2:** Cost of modeled lowest-cost nutrient adequate diets, when nutrient contents in foods were given as total dietary amounts (total nutrient diets) or bioavailable amounts (bioavailable nutrient diets), for different population groups in the United States

	Energy value of diet (kcal)	Total nutrient diets		Bioavailable nutrient diets	
		Cost of diet per day (United States $)	Daily cost per 1000 kcal of diet (United States $/1000 kcal)	Cost of diet per day (United States $)	Daily cost per 1000 kcal of diet (United States $/1000 kcal)
Children aged 1-3 y	1000	0.74	0.74	3.81	3.81
Children aged 4-8 y	1500	0.75	0.50	5.25	3.50
Male children aged 9-13 y	2000	0.81	0.40	3.11	1.55
Male adolescents aged 14-18 y	2700	1.11	0.41	4.21	1.56
Male adults aged 19-50 y	2600	1.12	0.43	2.99	1.15
Male adults aged >50 y	2300	1.09	0.48	3.15	1.37
Female children aged 9-13 y	1800	0.73	0.41	3.30	1.83
Female adolescents aged 14-18 y	2100	0.84	0.40	7.59	3.61
Female adults aged 19-50 y	2100	0.84	0.40	No Feasible Solution^1^	
Female adults aged >50 y	1800	0.87	0.48	1.66	0.92

1 It was not feasible to have bioavailable nutrient diets for female adults aged 19 to 50 y, when nutrient contents in foods were given as bioavailable amounts.

3. In the Results section, for Bioavailable nutrient diets: modeling lowest-cost nutrient adequate diets for different United States population groups, when nutrient contents in foods were given as bioavailable amounts, we apologize that several data errors were noted as a result of incorrect values used for calcium amounts for the beef food group in the foods databases.

Based on the re-analysis of data after correcting for calcium values, the following changes have been made:

3.1. Figure 1B has been revised to correct for the composition of the bioavailable nutrient diets for the 9 United States population groups, where feasible solutions were obtained. The new Figure 1 is appended below.
3.2. The following sentences have been corrected to: “Beef meat (4.5–170.0 g) was selected for all of the population groups, with the addition of beef liver for male adults aged >50 y (7.6 g) and female adults aged >50 y (4.8 g) (Figure 1B). Bioavailable nutrient diets included beef meat, beef liver, milk, eggs, pork liver sausages, fish, clams, and chicken, as foods sourced from animals (Figure 1B). Foods that originated from plants were legumes, nuts, seeds, vegetables (mushrooms, spinach, and hot chili peppers), fruits, grains (oats, wheat-based cereals, breakfast ready-to-eat cereals low and high in sugar, and corn grits), sauces and dressings (mayonnaise and soy sauce), and fats and oils (vegetable oils and margarine) (Figure 1B).”

**FIGURE 1:**
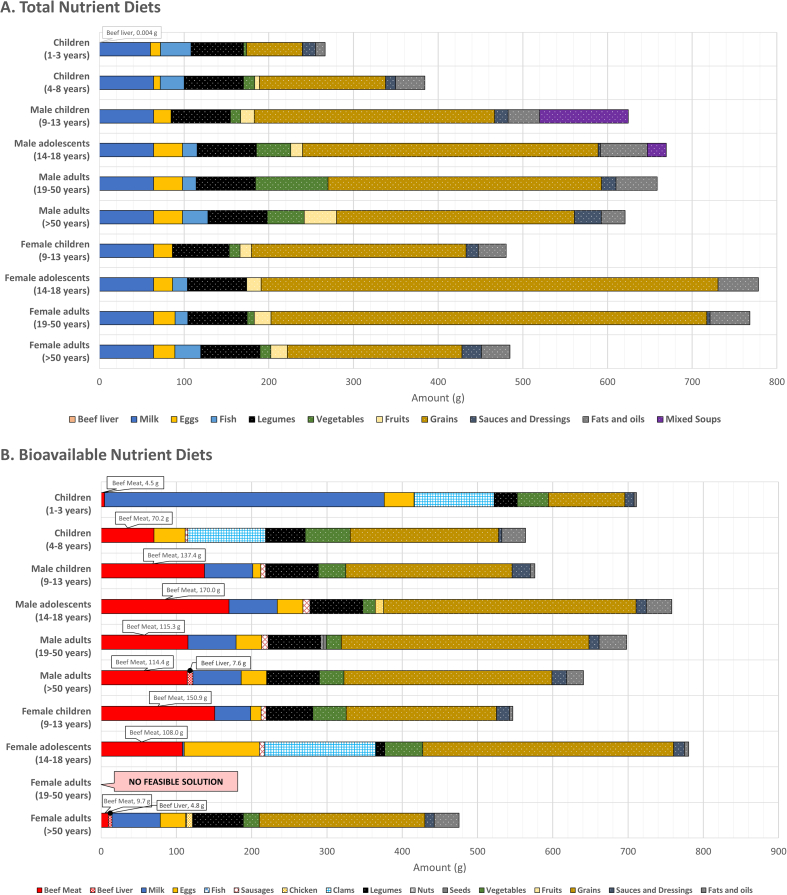
Composition of the modeled lowest-cost nutrient adequate diets for different population groups in the United States, when nutrient contents in foods were given as total dietary amounts ([A] total nutrient diets) or as bioavailable amounts ([B] bioavailable nutrient diets). (B) Bioavailable nutrient diets: for female adults aged 19 to 50 y, a feasible solution was not found when dietary contents of protein, vitamins, iron, and zinc were expressed on a bioavailable basis.

3.3. Table 2 as appended above has been updated to show the correct cost of the bioavailable nutrient diets for children aged 1 to 3 y, children aged 4 to 8 y, female children aged 9 to 13 y, female adolescents aged 14 to 18 y, and female adults aged >50 y.
3.4. The following sentence has been corrected to: “The daily diet cost of the bioavailable nutrient diets was higher compared with the total nutrient diets, ranging from United States $1.66 for female adults aged >50 y to United States $7.59 for female adolescents aged 14 to 18 y (Table 2).”
3.5. The following sentence has been corrected to: “Beef liver accounted for 2.7% and 3.3% of total diet cost, relative to the amount of beef liver in the bioavailable nutrient diets for male adults aged >50 y (7.6 g, 1.2% of total food weight) and female adults aged >50 y (4.8 g, 1.0 % of total food weight, respectively (data not shown).”
3.6. Figure 2 has been revised to correct for the cost and amount of beef meat in the bioavailable nutrient diets. The new Figure 2 is appended below.

**FIGURE 2:**
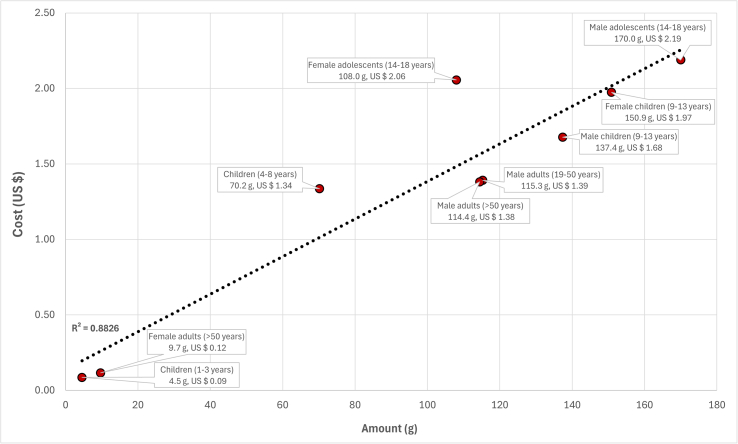
The cost (United States $) and amount (g) of beef meat in the modeled lowest-cost dietary patterns (bioavailable nutrient diets), when nutrient contents in foods were given as bioavailable amounts.

3.7. The following sentence has been corrected to: “Beef meat contributed between 2.3% of total diet cost for children aged 1 to 3 y, to 59.9% for female children aged 9 to 13 y, which was proportional to beef meat amounting between 0.6% of total food weight for children aged 1 to 3 y and 27.6% of total food weight for female children aged 9 to 13 y, respectively (data not shown).”
3.8. The following sentence has been corrected to: “The daily cost per 1000 kcal of the bioavailable nutrient diets was lowest at United States $0.92 for female adults aged >50 y and highest at United States $3.81 for children aged 1 to 3 y (Table 2).”
3.9. Table 4 has been corrected to show the first-limiting nutrients that were met at their daily minimum required levels (100%) for all of the 9 feasible United States population groups. The new Table 4 is appended below.

**Table 4:** Nutrients provided by the bioavailable nutrient diets, when nutrient contents in foods were given as bioavailable amounts, that were met at their daily minimum intake requirements (first-limiting) for different population groups in the United States^1^.

First-limiting nutrients in the bioavailable nutrient diets													
**Children aged 1-3 y**	Linoleic acid	α-linolenic acid	Dietary Fiber	Choline	Vitamin A	-	Vitamin D	Vitamin K	Calcium	Iron	Potassium	-	Zinc
**Children aged 4-8 y**	Linoleic acid	α-linolenic acid	Dietary Fiber	Choline	Vitamin A	-	-	Vitamin K	Calcium	Iron	Potassium	-	Zinc
**Male children aged 9-13 y**	Linoleic acid	α-linolenic acid	Dietary Fiber	Choline	Vitamin A	-	Vitamin D	Vitamin K	-	Iron	-	Sodium	Zinc
**Male adolescents aged 14-18 y**	Linoleic acid	α-linolenic acid	-	-	Vitamin A	Vitamin C	Vitamin D	Vitamin K	-	Iron	-	-	Zinc
**Male adults aged 19-50 y**	Linoleic acid	α-linolenic acid	-	Choline	Vitamin A	-	Vitamin D	Vitamin K	-	Iron	Potassium	Sodium	Zinc
**Male adults aged >50 y**	Linoleic acid	α-linolenic acid	-	Choline	Vitamin A	-	-	Vitamin K	-	Iron	Potassium	Sodium	Zinc
**Female children aged 9-13 y**	Linoleic acid	α-linolenic acid	Dietary Fiber	Choline	Vitamin A	-	Vitamin D	Vitamin K	Calcium	Iron	-	Sodium	Zinc
**Female adolescents aged 14-18 y**	Linoleic acid	α-linolenic acid	-	-	Vitamin A	Vitamin C	Vitamin D	Vitamin K	-	Iron	Potassium	-	Zinc
**Female adults aged 19-50 y**	No Feasible Solution^2^												
**Female adults aged >50 y**	Linoleic acid	α-linolenic acid	-	-	Vitamin A	-	Vitamin D	Vitamin K	-	Iron	Potassium	Sodium	Zinc

1 The contents of vitamin A, vitamin C, vitamin D, vitamin K, iron, and zinc, in foods were given as bioavailable amounts.

2 For female adults aged 19-50 y, a feasible solution was not found.

3.10. The following sentences have been corrected to: “Similarly, for both male adults population groups, choline, potassium, and sodium were first-limiting, but bioavailable vitamin D was supplied at its minimum required level for male adults aged 19 to 50 y, and close to requirements (102%) for male adults aged >50 y. Other nutrients that became first-limiting for female children aged 9 to 13 y were dietary fiber, choline, bioavailable vitamin D, calcium, and sodium. Bioavailable vitamin C, bioavailable vitamin D, and potassium were first-limiting for female adolescents aged 14 to 18 y, and the requirements for bioavailable vitamin D and potassium were met at minimum for female adults aged >50 y.”
3.11. The following sentences have been corrected to: “For United States children aged 1 to 3 y, a small portion of beef meat (4.5 g) in the bioavailable nutrient diets provided choline to 2.3% of requirement, and bioavailable zinc to 5.2% of requirement. Similarly, for United States female children aged 9 to 13 y, a bigger portion of beef meat (150.9 g) supplied choline (35.2% of requirement), bioavailable iron (10.1% of requirement), and bioavailable zinc (54.3% of requirement). For United States male adolescents aged 14 to 18 y, a bigger serving of beef meat (170.0 g) in the bioavailable nutrient diets provided nearly half of the RDA requirement for first-limiting bioavailable zinc (44.2%), and less than 10% of requirement for first-limiting linoleic acid (6.6%), and bioavailable iron (8.2 %). For United States male adults aged >50 y, beef meat (114.4 g) contributed choline (17.8% of requirement), bioavailable iron (7.4% of requirement), potassium (8.8% of requirement), and bioavailable zinc (29.0% of requirement), while beef liver (7.6 g) was the greatest contributor to bioavailable vitamin A (59.3% of requirement). For United States female adults aged >50 y, beef meat (9.7 g) supplied bioavailable zinc to 3.4% of requirement, and beef liver (4.8 g) contributed bioavailable vitamin A to 48.2% of requirement.”
4. In the Results section, for Diets for United States adults: modeling lowest-cost nutrient adequate diets for the representative United States adult population group, there were errors for the modeled diets due to incorrect values used for calcium amounts for the beef food group in the foods databases.

Based on the re-analysis of data after correcting for calcium values, the following changes have been made:

4.1. The following sentence has been corrected to: “On the basis of bioavailable nutrient contents in foods, the composition of the bioavailable nutrient diet was more varied with 28.9% of animal-sourced foods, including beef meat (13.4%), milk (0.2%), pork liver sausages (2.3%), eggs (13.0%), and clams (0.7%), and 71.1% of plant-based foods (Figure 3).”
4.2. Figure 3 has been revised to correct for the composition of the bioavailable nutrient diet for the representative adult population group in the United States. The new Figure 3 is appended below.
4.3. The following sentences have been corrected to: “Animal-sourced foods accounted for 51.2% of total diet cost, notably 38.5% for beef meat in the bioavailable nutrient diet, compared with 27.0% of total diet cost contributed by animal-sourced foods in the total nutrient diet (data not shown). The total nutrient diet provided daily 2350 kcal (9832.4 kJ), 331.4 g of carbohydrate, 77.9 g of fat, and 80.9 g of protein, and the energy proportions derived from carbohydrate (56.4%), fat (29.8%), and protein (13.8%) were within the AMDR values of 45% to 65% for carbohydrate, 20% to 35% for fat, and 10% to 35% for protein, respectively [41]. Similarly, the bioavailable nutrient diet supplied 346.5 g of carbohydrate, 66.1 g of fat, and 92.3 g of protein, with respective energy contributions of 59.0%, 25.3%, and 15.7%.”

**FIGURE 3:**
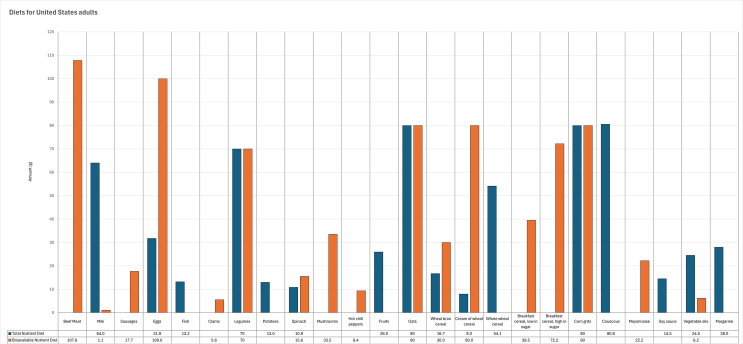
Composition of the modeled lowest-cost nutrient adequate diets for the representative adult population group in the United States (diets for United States adults), when nutrient contents in foods were given as total dietary amounts (total nutrient diet) or as bioavailable amounts (bioavailable nutrient diet).

4.4. The following sentences have been corrected to: “Dietary fiber was found to be nearly first-limiting (101% of requirement) for the total nutrient diet, but was sufficiently provided (129% of requirement) by the bioavailable nutrient diet, whereas protein was supplied in excess of requirement, with 159% and 181% for the total nutrient diet and bioavailable nutrient diet, respectively.”
4.5. Figure 4 has been revised to correct for the proportion of required vitamins and minerals provided by the modeled diets for the representative adult population group in the United States and contributed by 100 g (3.5 oz) of beef meat. The new Figure 4 is appended below.

**FIGURE 4:**
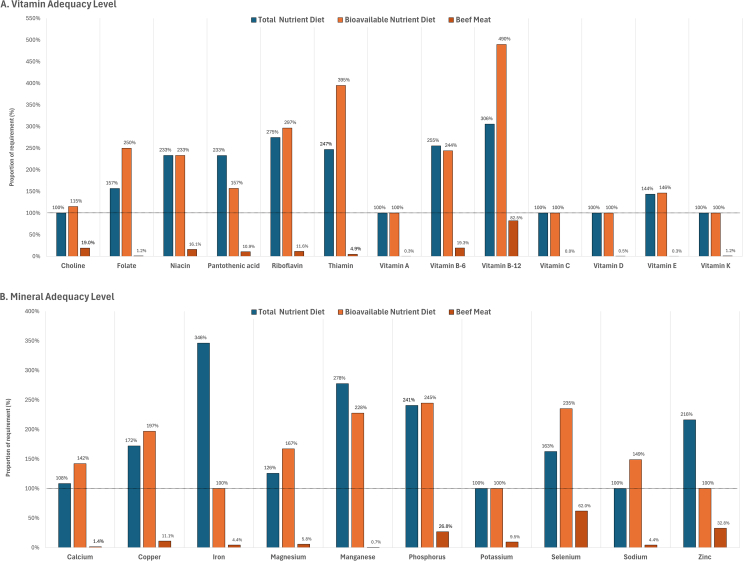
Proportion of required vitamins ([A] vitamin adequacy level) and minerals ([B] mineral adequacy level), provided by the modeled lowest-cost nutrient adequate diets for the representative adult population group in the United States, when nutrient contents in foods were given as total dietary amounts (total nutrient diet) or as bioavailable amounts (bioavailable nutrient diet). The contribution of 100 g (3.5 oz) of beef meat to daily vitamin and mineral requirements is also reported (beef meat).

4.6. The following sentence has been corrected to: “For United States adults, beef meat (102.8 g, 3.6 oz, 13.4% of total food weight) included in the bioavailable nutrient diet provided the first-limiting bioavailable zinc to 34.6% of RDA requirement, the first-limiting potassium to 9.9 % of AI requirement, and the first-limiting bioavailable iron to 4.6% of RDA requirement. A serving of 100 g (3.5 oz) of cooked beef meat contributed protein to 55.6% of daily requirement, vitamin B-12 to 82.5% of requirement, selenium to 62.0% of requirement, zinc to 32.8% of requirement, phosphorus to 26.8% of requirement, and also provided other essential vitamins and minerals, as presented in Figure 4.”
4.7. The following sentences have been corrected to: “The shadow prices indicated that decreasing the daily minimum intake requirements for linoleic acid, α-linolenic acid, choline, vitamin A, vitamin C, vitamin D, vitamin K, potassium, and sodium would result in a reduction in daily diet cost. Bioavailable iron and bioavailable zinc had the highest shadow prices (–0.954 and –0.652, respectively), to predict a possible reduction in daily diet cost (United States $4.39 for the bioavailable nutrient diet) per unit decrease in the daily recommended intake requirements for iron and zinc.”
5. In the Discussion section, the following sentences have been corrected to:

5.1. “When bioavailability was considered for dietary contents of protein, vitamins, iron, and zinc, a greater variety of animal-sourced foods was selected to be part of modelled lowest-cost diets, including milk, eggs, fish, clams, sausages, chicken, beef liver, and beef meat.”
5.2. “Children aged 4 to 8 y and male and female adolescents aged 14 to 18 y faced the highest diet costs, and children aged 1 to 3 y had the highest diet cost per 1000 kcal.”
5.3. “Beef meat was the lowest-cost contributor to bioavailable protein, bioavailable vitamin B-12, and other B-group vitamins, phosphorus, selenium, and bioavailable zinc.”

6. In the Supplementary data to this article, there were inaccurate values in Supplemental Table 8 and Supplemental Table 9.

6.1. Supplemental Table 8 has been corrected to show how the requirements for nutrients were fulfilled by the bioavailable nutrient diets on a dietary bioavailable nutrient basis. The new Supplemental Table 8 is appended below.

**Supplemental Table 8:** Proportion of required macronutrients, vitamins, and minerals, provided by the modelled lowest-cost nutrient adequate diets, the Bioavailable Nutrient Diets when nutrient contents in foods were given as bioavailable amounts, for different population groups in the United States^1^.

Bioavailable Nutrient Diets	Linoleic acid (%)	α-linolenic acid (%)	Dietary Fiber (%)	Protein (%)
Children aged 1 to 3 years	100	100	100	382
Children aged 4 to 8 years	100	100	100	369
Male children aged 9 to 13 years	100	100	100	268
Male adolescents aged 14 to 18 years	100	100	105	222
Male adults aged 19 to 50 years	100	100	106	176
Male adults aged over 50 years	100	100	121	168
Female children aged 9 to 13 years	100	100	100	259
Female adolescents aged 14 to 18 years	100	100	107	216
Female adults aged 19 to 50 years	No Feasible Solution^2^			
Female adults aged over 50 years	100	100	145	131

	Choline (%)	Folate (%)	Niacin (%)	Pantothenic acid (%)	Riboflavin (%)	Thiamin (%)	Vitamin A (%)	Vitamin B-6 (%)	Vitamin B-12 (%)	Vitamin C (%)	Vitamin D (%)	Vitamin E (%)	Vitamin K (%)
Children aged 1 to 3 years	100	107	167	206	304	271	100	196	3449	204	100	138	100
Children aged 4 to 8 years	100	200	187	130	261	410	100	206	2556	110	123	136	100
Male children aged 9 to 13 years	100	200	167	183	254	294	100	216	471	107	100	125	100
Male adolescents aged 14 to 18 years	117	200	187	173	248	336	100	278	535	100	100	187	100
Male adults aged 19 to 50 years	100	224	219	234	283	305	100	350	623	114	100	237	100
Male adults aged over 50 years	100	242	219	211	290	297	100	246	701	102	102	199	100
Female children aged 9 to13 years	100	194	167	193	247	332	100	210	466	103	100	106	100
Female adolescents aged 14 to 18 years	116	200	214	157	305	398	100	207	1964	100	100	113	100
Female adults aged 19 to 50 years	No Feasible Solution^2^												
Female adults aged over 50 years	116	250	250	242	354	357	100	265	583	146	100	207	100

	Calcium (mg)	Copper (μg)	Iron (mg)	Magnesium (mg)	Manganese (mg)	Phosphorus (mg)	Potassium (mg)	Selenium (μg)	Sodium (mg)	Zinc (mg)
Children aged 1 to 3 years	100	294	100	441	118	277	100	352	106	100
Children aged 4 to 8 years	100	332	100	318	188	242	100	338	150	100
Male children aged 9 to 13 years	112	201	100	221	184	138	132	271	100	100
Male adolescents aged 14 to 18 years	139	187	100	162	400	181	127	286	129	100
Male adults aged 19 to 50 years	206	178	100	175	309	314	100	288	100	100
Male adults aged over 50 years	170	301	100	171	310	314	100	275	100	100
Female children aged 9 to 13 years	100	181	100	203	217	125	127	242	100	100
Female adolescents aged 14 to 18 years	130	180	100	129	330	135	100	271	153	100
Female adults aged 19 to 50 years	No Feasible Solution^2^									
Female adults aged over 50 years	182	208	100	170	179	248	100	109	100	100

1 The contents of protein, folate, niacin, pantothenic acid, riboflavin, thiamin, vitamin A, vitamin B-6, vitamin B-12, vitamin C, vitamin D, vitamin E, vitamin K, iron, and zinc, in foods were given as bioavailable amounts, by factoring in published bioavailability data estimates on total dietary gross amounts.

2 It was not feasible to have data for female adults aged 19 to 50 years, based on the constraints imposed on the diet model and when dietary contents of protein, vitamins, iron, and zinc were expressed on a bioavailable basis.

6.2. Supplemental Table 9 has been revised to show the corrected shadow prices resulting from sensitivity analyses for the bioavailable nutrient diet for the representative adult population group in the United States. The new Supplemental Table 9 is appended below.

**Supplemental Table 9:** Sensitivity analyses for modelled lowest-cost dietary patterns, when nutrient contents in foods were given as total dietary amounts (Total Nutrient Diet) or bioavailable amounts (Bioavailable Nutrient Diet), for the representative adult population in the United States. The shadow prices represent the potential decrease in daily minimum cost of modelled diets (US $ 0.96 for Total Nutrient Diet and US $ 4.39 for Bioavailable Nutrient Diet, respectively) per unit decrease or increase in daily recommended intake requirements of nutrients.

	Total Nutrient Diet		Bioavailable Nutrient Diet	
		Shadow price		Shadow price
Linoleic acid	< 14.5 g	-0.00099	< 14.5 g	-0.05226
α-linolenic acid	< 1.35 g	-0.00037	< 1.35 g	-0.03392
Choline	< 487.5 mg	-0.00016		
Folate			> 1000 mg	-0.00002
Niacin	> 35 mg	-0.00090	> 35 mg	-0.21002
Vitamin A	< 800 μg	-0.00001	< 800 μg	-0.00010
Vitamin C	< 82.5 mg	-0.00053	< 82.5 mg	-0.00418
Vitamin D	< 15 μg	-0.02822	< 15 μg	-0.04759
Vitamin K	< 105 μg	-0.00095	< 105 μg	-0.00105
Iron	> 45 mg	-0.00054	< 13 mg	-0.95440
Potassium	< 3000 mg	-0.00007	< 3000 mg	-0.00029
Sodium	< 1500 mg	-0.00010		
Zinc			< 9.5 mg	-0.65191

These corrections do not change the conclusions of the article. The authors apologize for any inconvenience caused.

