# Supplementary material for: The Role of Beef for the Lowest Cost and Adequate Provision of Bioavailable Nutrients in Modeled Diets at a Population Level in the United States
Source: Curr Dev Nutr. 2025 Nov 26;9(12):107604. doi: 10.1016/j.cdnut.2025.107604 (PMC12765099; doi:10.1016/j.cdnut.2025.107604)
Supplement: Multimedia component 1 [file mmc1.docx]

**Supplemental Table 1.** List of foods included in the Linear Programming diet models ($N_{f}$ = 1977)^1^ categorized into 27 main food groups.

| **Food source** | **Food group** | **Food subgroup** | **Number of foods** |
| --- | --- | --- | --- |
| ***Animal*** |  |  | ***733*** |
|  | Beef | Composite beef, beef steak, beef roast, beef pot roast, beef ribs, corned beef, beef brisket, ground beef, beef liver, beef bacon, beef jerky | 144 |
|  | Milk | Dairy milk, buttermilk, dried milk, evaporated milk, goat milk | 24 |
|  | Dairy products | Butter, cheese, cream, sour cream, yogurt, ice cream | 95 |
|  | Pork | Composite pork, pork roast, pork steak, pork loin, pork chop, pork ribs, pork ham, ground pork, pork skins | 131 |
|  | Lamb | Composite lamb, lamb leg and loin roast, lamb loin chop, lamb ribs, ground lamb | 42 |
|  | Venison | Venison steak, venison roast, ground venison | 7 |
|  | Sausages, cold cuts, and cured meats | Sausages, frankfurters, bologna, deli meats, salami, luncheon meats, ham, bacon, pate | 67 |
|  | Chicken | Chicken meats, chicken breast, chicken drumstick, chicken thigh, chicken leg, chicken wing, ground chicken, chicken liver, chicken nuggets and patties | 79 |
|  | Eggs | Whole eggs, egg whites, egg yolks | 8 |
|  | Turkey | Turkey meats, turkey drumstick, turkey thigh, turkey wing, ground turkey | 24 |
|  | Fish | Fresh raw fish, cooked fish, canned fish, frozen fish sticks | 77 |
|  | Seafood | Clam, abalone, mussel, scallop, squid, octopus, crab, crayfish, lobster, shrimp | 35 |
| ***Plant*** |  |  | ***1200*** |
|  | Legumes | White beans, black beans, fava beans, lima beans, pink beans, pinto beans, kidney beans, mung beans, cowpeas, chickpeas, hummus, falafel, split peas, lentils, baked beans, refried beans, soybeans | 74 |
|  | Plant-based milk and dairy substitutes | Soymilk, almond milk, rice milk, coconut milk, imitation cheese, cream substitute | 17 |
|  | Nuts | Almonds, almond butter and paste, Brazil nuts, cashew nuts, cashew butter, chestnuts, dried coconut meat, coconut milk and cream, hazelnuts, macadamia nuts, pecans, pine nuts, pistachio nuts, peanuts, peanut butter, mixed nuts, trail mix | 80 |
|  | Seeds | Chia seeds, flax seeds, sunflower seeds, sesame seeds, sesame butter | 22 |
|  | Vegetables | Artichokes, asparagus, green beans, beets, broccoli, brussels sprouts, cabbage, cactus, cauliflower, celery, fennel, sweet corn, cucumber, pickles, edamame, eggplant, yambean, kohlrabi, leeks, lettuce, mushrooms, onions, green peas, sweet peppers, hot chili peppers, radishes, turnips, rutabagas, podded peas, pumpkin, squash, sprouts, turnip greens, collards, kale, spinach, carrots, tomatoes, plantains, sweet potatoes, potatoes, vegetable chips, garlic, yeast, seaweed, frozen mixed vegetables, vegetable juices | 313 |
|  | Fruits | Apples, apricots, avocados, bananas, melons, cherries, figs, grapes, kiwifruit, lychee, mango, papaya, nectarine, peach, passion fruit, pear, persimmon, pineapple, plum, pomegranate, soursop, oranges, mixed citrus fruits, tamarind, mixed berries, dried fruits, fruit juices, canned fruits | 190 |
|  | Breakfast cereal | Ready-to-eat cereal, low in sugar (=<21.2 g/100g), ready-eat cereal, high in sugar (>21.2 g/100g), cooked whole wheat, wheat bran, oatmeal, cream of wheat | 165 |
|  | Cereal grains | Corn grits, hominy grits, cornmeal, millet, wheat germ, barley, buckwheat groats, bulgur, couscous, quinoa | 35 |
|  | Grains | Yeast breads, bread rolls, bagels, other baked products, wheat flour, stuffing, tortillas, taco shells | 122 |
|  | Rice | White rice, brown rice | 20 |
|  | Pasta and noodles | Pasta, vegetable pasta, whole grain pasta, gluten free pasta, egg noodles, rice noodles | 37 |
|  | Sweets | Sugar, sugar substitute, honey, molasses, syrup, jam, jelly, topping | 35 |
|  | Sauces and dressings | Mayonnaise, salad dressings, sauces, soy-based sauces, gravy | 64 |
|  | Fats and oils | Vegetable oils, margarine | 26 |
| ***Mixed*** | Soups | Animal-based soups, animal-based broths, plant-based soups, mixed animal- and plant-based soups | ***44*** |

^1^ $N_{f}$ is the number of foods included in the Linear Programming diet models.

Data sourced from United States Department of Agriculture (1,2).

**Supplemental Table 2.** Contribution of energy from macronutrient and daily minimum intake requirements for fatty acids, dietary fiber, and protein, recommended as acceptable for different population groups in the United States.

|  | **Carbohydrate (% energy)** | **Fat**  **(% of energy)** | **Protein**  **(% of energy)** | **Linoleic acid (g)** | **α-linolenic acid (g)** | **Dietary Fiber (g)** | **Protein (g)** |
| --- | --- | --- | --- | --- | --- | --- | --- |
| Children aged 1 to 3 years | 45-65 | 30-40 | 5-20 | 7 | 0.7 | 19 | 13 |
| Children aged 4 to 8 years | 45-65 | 25-35 | 10-30 | 10 | 0.9 | 25 | 19 |
| Male children aged 9 to 13 years | 45-65 | 25-35 | 10-30 | 12 | 1.2 | 31 | 34 |
| Male adolescents aged 14 to 18 years | 45-65 | 25-35 | 10-30 | 16 | 1.6 | 38 | 52 |
| Male adults aged 19 to 50 years | 45-65 | 20-35 | 10-35 | 17 | 1.6 | 38 | 56 |
| Male adults aged over 50 years | 45-65 | 20-35 | 10-35 | 14 | 1.6 | 30 | 56 |
| Female children aged 9 to 13 years | 45-65 | 25-35 | 10-30 | 10 | 1.0 | 26 | 34 |
| Female adolescents aged 14 to 18 years | 45-65 | 25-35 | 10-30 | 11 | 1.1 | 26 | 46 |
| Female adults aged 19 to 50 years | 45-65 | 20-35 | 10-35 | 12 | 1.1 | 25 | 46 |
| Female adults aged over 50 years | 45-65 | 20-35 | 10-35 | 11 | 1.1 | 21 | 46 |

Data sourced from Institute of Medicine (3,4) and National Academies of Sciences, Engineering, and Medicine (5).

**Supplemental Table 3.** Daily minimum intake requirements for vitamins, recommended for different population groups in the United States.

|  | **Choline (mg)** | **Folate (µg)^1^** | **Niacin (mg)^2^** | **Pantothenic acid (mg)** | **Riboflavin (mg)** | **Thiamin (mg)** | **Vitamin A (µg)^3^** | **Vitamin B-6 (mg)** | **Vitamin B-12 (µg)** | **Vitamin C (mg)** | **Vitamin D (µg)^4^** | **Vitamin E (mg)^5^** | **Vitamin K (µg)^6^** |
| --- | --- | --- | --- | --- | --- | --- | --- | --- | --- | --- | --- | --- | --- |
| Children aged 1 to 3 years | 200 | 150 | 6 | 2 | 0.5 | 0.5 | 300 | 0.5 | 0.9 | 15 | 15 | 6 | 30 |
| Children aged 4 to 8 years | 250 | 200 | 8 | 3 | 0.6 | 0.6 | 400 | 0.6 | 1.2 | 25 | 15 | 7 | 55 |
| Male children aged 9 to 13 years | 375 | 300 | 12 | 4 | 0.9 | 0.9 | 600 | 1.0 | 1.8 | 45 | 15 | 11 | 60 |
| Male adolescents aged 14 to 18 years | 550 | 400 | 16 | 5 | 1.3 | 1.2 | 900 | 1.3 | 2.4 | 75 | 15 | 15 | 75 |
| Male adults aged 19 to 50 years | 550 | 400 | 16 | 5 | 1.3 | 1.2 | 900 | 1.3 | 2.4 | 90 | 15 | 15 | 120 |
| Male adults aged over 50 years | 550 | 400 | 16 | 5 | 1.3 | 1.2 | 900 | 1.7 | 2.4 | 90 | 17.5 | 15 | 120 |
| Female children aged 9 to 13 years | 375 | 300 | 12 | 4 | 0.9 | 0.9 | 600 | 1.0 | 1.8 | 45 | 15 | 11 | 60 |
| Female adolescents aged 14 to 18 years | 400 | 400 | 14 | 5 | 1.0 | 1.0 | 700 | 1.2 | 2.4 | 65 | 15 | 15 | 75 |
| Female adults aged 19 to 50 years | 425 | 400 | 14 | 5 | 1.1 | 1.1 | 700 | 1.3 | 2.4 | 75 | 15 | 15 | 90 |
| Female adults aged over 50 years | 425 | 400 | 14 | 5 | 1.1 | 1.1 | 700 | 1.5 | 2.4 | 75 | 17.5 | 15 | 90 |

^1^Folate is expressed as dietary folate equivalents (DFE), whereby 1 µg of DFE equals 1 µg of food folate, or 0.6 µg of folic acid from fortified foods or as a dietary supplement consumed with food.

^2^Niacin is expressed as niacin equivalents (NE), whereby 1 mg of niacin is equivalent to 60 mg of tryptophan.

^3^Vitamin A is expressed as retinol activity equivalents (RAE), whereby 1 µg of RAE equals 1 µg of retinol, 12 µg of β-carotene, 24 µg of other pro-vitamin A carotenoids.

^4^Vitamin D is the sum of cholecalciferol (vitamin D-3) and ergocalciferol (vitamin D-2).

^5^Vitamin E is expressed as α-tocopherol.

^6^Vitamin K is expressed as phylloquinone.

Data sourced from Institute of Medicine (3,6-10).

**Supplemental Table 4.** Daily minimum intake requirements for minerals, recommended for different population groups in the United States.

|  | **Calcium (mg)** | **Copper (µg)** | **Iron (mg)** | **Magnesium (mg)** | **Manganese (mg)** | **Phosphorus (mg)** | **Potassium (mg)** | **Selenium (µg)** | **Sodium (mg)** | **Zinc (mg)** |
| --- | --- | --- | --- | --- | --- | --- | --- | --- | --- | --- |
| Children aged 1 to 3 years | 700 | 340 | 7 | 80 | 1.2 | 460 | 2000 | 20 | 800 | 3 |
| Children aged 4 to 8 years | 1000 | 440 | 10 | 130 | 1.5 | 500 | 2300 | 30 | 1000 | 5 |
| Male children aged 9 to 13 years | 1300 | 700 | 8 | 240 | 1.9 | 1250 | 2500 | 40 | 1200 | 8 |
| Male adolescents aged 14 to 18 years | 1300 | 890 | 11 | 410 | 2.2 | 1250 | 3000 | 55 | 1500 | 11 |
| Male adults aged 19 to 50 years | 1000 | 900 | 8 | 410 | 2.3 | 700 | 3400 | 55 | 1500 | 11 |
| Male adults aged over 50 years | 1100 | 900 | 8 | 420 | 2.3 | 700 | 3400 | 55 | 1500 | 11 |
| Female children aged 9 to 13 years | 1300 | 700 | 8 | 240 | 1.6 | 1250 | 2300 | 40 | 1200 | 8 |
| Female adolescents aged 14 to 18 years | 1300 | 890 | 15 | 360 | 1.6 | 1250 | 2300 | 55 | 1500 | 9 |
| Female adults aged 19 to 50 years | 1000 | 900 | 18 | 315 | 1.8 | 700 | 2600 | 55 | 1500 | 8 |
| Female adults aged over 50 years | 1100 | 900 | 8 | 320 | 1.8 | 700 | 2600 | 55 | 1500 | 8 |

Data sourced from Institute of Medicine (3,6-8,10,11) and National Academies of Sciences, Engineering, and Medicine (12).

**Supplemental Table 5.** Daily maximum (tolerable upper) intake levels for vitamins, recommended for different population groups in the United States.

|  | **Choline (mg)** | **Folate (µg)^1^** | **Niacin (mg)^1^** | **Vitamin A (µg)^2^** | **Vitamin B-6 (mg)** | **Vitamin C (mg)** | **Vitamin D (µg)** | **Vitamin E (mg)^1^** |
| --- | --- | --- | --- | --- | --- | --- | --- | --- |
| Children aged 1 to 3 years | 1000 | 300 | 10 | 600 | 30 | 400 | 63 | 200 |
| Children aged 4 to 8 years | 1000 | 400 | 15 | 900 | 40 | 650 | 75 | 300 |
| Children aged 9 to 13 years | 2000 | 600 | 20 | 1700 | 60 | 1200 | 100 | 600 |
| Adolescents aged 14 to 18 years | 3000 | 800 | 30 | 2800 | 80 | 1800 | 100 | 800 |
| Adults aged over 18 years | 3500 | 1000 | 35 | 3000 | 100 | 2000 | 100 | 1000 |

^1^The daily maximum intake levels for folate, niacin, and vitamin E, apply to synthetic forms obtained from fortified foods or supplements consumed with fortified foods.

^2^ The daily maximum intake levels for vitamin A apply to pre-formed vitamin A retinol only, whereby 1 µg of retinol equals 1 µg of RAE.

Data sourced from Institute of Medicine (3,6-9).

**Supplemental Table 6.** Daily maximum (tolerable upper) intake levels for minerals, recommended for different population groups in the United States.

|  | **Calcium (mg)** | **Copper (µg)** | **Iron (mg)** | **Manganese (mg)** | **Phosphorus (mg)** | **Selenium (µg)** | **Sodium (mg)^1^** | **Zinc (mg)** |
| --- | --- | --- | --- | --- | --- | --- | --- | --- |
| Children aged 1 to 3 years | 2500 | 1000 | 40 | 2 | 3000 | 90 | 1200 | 7 |
| Children aged 4 to 8 years | 2500 | 3000 | 40 | 3 | 3000 | 150 | 1500 | 12 |
| Children aged 9 to 13 years | 3000 | 5000 | 40 | 6 | 4000 | 280 | 1800 | 23 |
| Adolescents aged 14 to 18 years | 3000 | 8000 | 45 | 9 | 4000 | 400 | 2300 | 34 |
| Adults aged over 18 years | 2500 | 10000 | 45 | 11 | 4000 | 400 | 2300 | 40 |

^1^The daily maximum intake levels for sodium, apply to the level of sodium intake that is likely to pose no risk of chronic disease.

Data sourced from Institute of Medicine (3,6-8,10,11) and National Academies of Sciences, Engineering, and Medicine (12).

**Supplemental Table 7.**  Proportion of required macronutrients, vitamins, and minerals, provided by the modelled lowest-cost nutrient adequate diets, the Total Nutrient Diets when nutrient contents in foods were given as total gross dietary amounts, for different population groups in the United States.

| **Total Nutrient Diets** | **Linoleic acid (%)** | **α-linolenic acid (%)** | **Dietary Fiber (%)** | **Protein (%)** |
| --- | --- | --- | --- | --- |
| Children aged 1 to 3 years | 100 | 100 | 100 | 372 |
| Children aged 4 to 8 years | 100 | 100 | 100 | 323 |
| Male children aged 9 to 13 years | 100 | 100 | 100 | 208 |
| Male adolescents aged 14 to 18 years | 106 | 100 | 100 | 177 |
| Male adults aged 19 to 50 years | 100 | 100 | 100 | 154 |
| Male adults aged over 50 years | 100 | 117 | 100 | 145 |
| Female children aged 9 to 13 years | 100 | 100 | 100 | 186 |
| Female adolescents aged 14 to 18 years | 100 | 146 | 115 | 159 |
| Female adults aged 19 to 50 years | 100 | 109 | 118 | 159 |
| Female adults aged over 50 years | 100 | 100 | 122 | 145 |

|  | **Choline (%)** | **Folate (%)** | **Niacin (%)** | **Pantothenic acid (%)** | **Riboflavin (%)** | **Thiamin (%)** | **Vitamin A (%)** | **Vitamin B-6 (%)** | **Vitamin B-12 (%)** | **Vitamin C (%)** | **Vitamin D (%)** | **Vitamin E (%)** | **Vitamin K (%)** |
| --- | --- | --- | --- | --- | --- | --- | --- | --- | --- | --- | --- | --- | --- |
| Children aged 1 to 3 years | 100 | 200 | 167 | 211 | 265 | 166 | 100 | 237 | 754 | 100 | 100 | 100 | 100 |
| Children aged 4 to 8 years | 100 | 200 | 187 | 190 | 280 | 221 | 118 | 191 | 460 | 100 | 100 | 100 | 100 |
| Male children aged 9 to 13 years | 100 | 200 | 167 | 198 | 254 | 270 | 100 | 151 | 225 | 100 | 100 | 100 | 100 |
| Male adolescents aged 14 to 18 years | 100 | 200 | 187 | 198 | 210 | 256 | 103 | 209 | 244 | 100 | 100 | 100 | 100 |
| Male adults aged 19 to 50 years | 100 | 250 | 219 | 215 | 238 | 343 | 120 | 251 | 256 | 100 | 100 | 100 | 100 |
| Male adults aged over 50 years | 100 | 241 | 219 | 214 | 261 | 255 | 168 | 210 | 331 | 100 | 100 | 100 | 100 |
| Female children aged 9 to 13 years | 100 | 200 | 167 | 195 | 260 | 286 | 100 | 150 | 238 | 100 | 100 | 101 | 100 |
| Female adolescents aged 14 to 18 years | 100 | 174 | 214 | 202 | 298 | 277 | 100 | 197 | 296 | 100 | 100 | 126 | 100 |
| Female adults aged 19 to 50 years | 100 | 169 | 250 | 217 | 304 | 266 | 100 | 246 | 313 | 100 | 100 | 139 | 100 |
| Female adults aged over 50 years | 100 | 191 | 250 | 203 | 306 | 314 | 100 | 201 | 368 | 100 | 100 | 126 | 100 |

|  | **Calcium (mg)** | **Copper (µg)** | **Iron (mg)** | **Magnesium (mg)** | **Manganese (mg)** | **Phosphorus (mg)** | **Potassium (mg)** | **Selenium (µg)** | **Sodium (mg)** | **Zinc (mg)** |
| --- | --- | --- | --- | --- | --- | --- | --- | --- | --- | --- |
| Children aged 1 to 3 years | 100 | 269 | 135 | 265 | 111 | 214 | 100 | 181 | 100 | 221 |
| Children aged 4 to 8 years | 100 | 254 | 202 | 239 | 198 | 256 | 100 | 142 | 100 | 180 |
| Male children aged 9 to 13 years | 100 | 204 | 447 | 169 | 295 | 132 | 101 | 179 | 100 | 179 |
| Male adolescents aged 14 to 18 years | 100 | 214 | 409 | 119 | 279 | 147 | 106 | 202 | 100 | 152 |
| Male adults aged 19 to 50 years | 107 | 183 | 502 | 117 | 226 | 254 | 100 | 182 | 100 | 152 |
| Male adults aged over 50 years | 103 | 185 | 558 | 109 | 205 | 238 | 100 | 199 | 100 | 161 |
| Female children aged 9 to 13 years | 100 | 175 | 456 | 152 | 313 | 124 | 101 | 172 | 100 | 178 |
| Female adolescents aged 14 to 18 years | 100 | 153 | 300 | 118 | 334 | 133 | 112 | 141 | 100 | 210 |
| Female adults aged 19 to 50 years | 100 | 145 | 250 | 133 | 274 | 222 | 100 | 142 | 100 | 254 |
| Female adults aged over 50 years | 100 | 127 | 562 | 118 | 198 | 209 | 100 | 135 | 100 | 236 |

**Supplemental Table 8.** Proportion of required macronutrients, vitamins, and minerals, provided by the modelled lowest-cost nutrient adequate diets, the Bioavailable Nutrient Diets when nutrient contents in foods were given as bioavailable amounts, for different population groups in the United States^1^.

| **Bioavailable Nutrient Diets** | **Linoleic acid (%)** | **α-linolenic acid (%)** | **Dietary Fiber (%)** | **Protein (%)** |
| --- | --- | --- | --- | --- |
| Children aged 1 to 3 years | 100 | 100 | 100 | 338 |
| Children aged 4 to 8 years | 100 | 100 | 100 | 378 |
| Male children aged 9 to 13 years | 100 | 100 | 100 | 270 |
| Male adolescents aged 14 to 18 years | 100 | 100 | 105 | 222 |
| Male adults aged 19 to 50 years | 100 | 100 | 107 | 176 |
| Male adults aged over 50 years | 100 | 100 | 121 | 168 |
| Female children aged 9 to 13 years | 100 | 100 | 100 | 255 |
| Female adolescents aged 14 to 18 years | 100 | 100 | 100 | 221 |
| Female adults aged 19 to 50 years | No Feasible Solution^2^ | | | |
| Female adults aged over 50 years | 100 | 100 | 141 | 125 |

|  | **Choline (%)** | **Folate (%)** | **Niacin (%)** | **Pantothenic acid (%)** | **Riboflavin (%)** | **Thiamin (%)** | **Vitamin A (%)** | **Vitamin B-6 (%)** | **Vitamin B-12 (%)** | **Vitamin C (%)** | **Vitamin D (%)** | **Vitamin E (%)** | **Vitamin K (%)** |
| --- | --- | --- | --- | --- | --- | --- | --- | --- | --- | --- | --- | --- | --- |
| Children aged 1 to 3 years | 100 | 113 | 167 | 156 | 228 | 280 | 100 | 175 | 2493 | 324 | 100 | 467 | 100 |
| Children aged 4 to 8 years | 100 | 200 | 188 | 141 | 263 | 405 | 100 | 209 | 2898 | 113 | 126 | 134 | 100 |
| Male children aged 9 to 13 years | 100 | 200 | 167 | 177 | 265 | 306 | 100 | 293 | 522 | 105 | 100 | 128 | 100 |
| Male adolescents aged 14 to 18 years | 117 | 200 | 188 | 173 | 248 | 336 | 100 | 278 | 535 | 100 | 100 | 187 | 100 |
| Male adults aged 19 to 50 years | 100 | 216 | 219 | 246 | 284 | 307 | 100 | 336 | 606 | 100 | 100 | 233 | 100 |
| Male adults aged over 50 years | 100 | 242 | 219 | 211 | 290 | 297 | 100 | 246 | 701 | 102 | 102 | 199 | 100 |
| Female children aged 9 to 13 years | 100 | 200 | 167 | 190 | 243 | 325 | 100 | 211 | 464 | 105 | 100 | 113 | 100 |
| Female adolescents aged 14 to 18 years | 106 | 190 | 214 | 172 | 299 | 396 | 100 | 209 | 2274 | 104 | 111 | 114 | 100 |
| Female adults aged 19 to 50 years | No Feasible Solution^2^ | | | | | | | | | | | | |
| Female adults aged over 50 years | 109 | 235 | 250 | 256 | 353 | 366 | 100 | 304 | 585 | 153 | 100 | 238 | 100 |

|  | **Calcium (mg)** | **Copper (µg)** | **Iron (mg)** | **Magnesium (mg)** | **Manganese (mg)** | **Phosphorus (mg)** | **Potassium (mg)** | **Selenium (µg)** | **Sodium (mg)** | **Zinc (mg)** |
| --- | --- | --- | --- | --- | --- | --- | --- | --- | --- | --- |
| Children aged 1 to 3 years | 100 | 294 | 100 | 457 | 151 | 204 | 100 | 322 | 150 | 100 |
| Children aged 4 to 8 years | 100 | 426 | 100 | 314 | 177 | 248 | 100 | 350 | 150 | 100 |
| Male children aged 9 to 13 years | 116 | 211 | 100 | 235 | 193 | 144 | 135 | 269 | 100 | 100 |
| Male adolescents aged 14 to 18 years | 137 | 187 | 100 | 162 | 400 | 181 | 127 | 286 | 130 | 100 |
| Male adults aged 19 to 50 years | 158 | 174 | 100 | 175 | 310 | 313 | 100 | 288 | 100 | 100 |
| Male adults aged over 50 years | 119 | 301 | 100 | 171 | 310 | 314 | 100 | 275 | 100 | 100 |
| Female children aged 9 to 13 years | 119 | 182 | 100 | 200 | 174 | 121 | 126 | 265 | 100 | 100 |
| Female adolescents aged 14 to 18 years | 100 | 250 | 100 | 124 | 330 | 133 | 100 | 275 | 153 | 100 |
| Female adults aged 19 to 50 years | No Feasible Solution^2^ | | | | | | | | | |
| Female adults aged over 50 years | 100 | 120 | 100 | 165 | 174 | 236 | 100 | 120 | 113 | 100 |

^1^The contents of protein, folate, niacin, pantothenic acid, riboflavin, thiamin, vitamin A, vitamin B-6, vitamin B-12, vitamin C, vitamin D, vitamin E, vitamin K, iron, and zinc, in foods were given as bioavailable amounts, by factoring in published bioavailability data estimates on total dietary gross amounts.

^2^It was not feasible to have data for female adults aged 19 to 50 years, based on the constraints imposed on the diet model and when dietary contents of protein, vitamins, iron, and zinc were expressed on a bioavailable basis.

**Supplemental Table 9.** Sensitivity analyses for modelled lowest-cost dietary patterns, when nutrient contents in foods were given as total dietary amounts (Total Nutrient Diet) or bioavailable amounts (Bioavailable Nutrient Diet), for the representative adult population group in the United States. The shadow prices represent the potential decrease in daily minimum cost of modelled diets (US $ 0.96 for Total Nutrient Diet and US $ 4.39 for Bioavailable Nutrient Diet, respectively) per unit decrease or increase in daily recommended intake requirements of nutrients.

|  | **Total Nutrient Diet** | | **Bioavailable Nutrient Diet** | |
| --- | --- | --- | --- | --- |
|  |  | **Shadow price** |  | **Shadow price** |
| Linoleic acid | < 14.5 g | -0.00099 | < 14.5 g | -0.05582 |
| α-linolenic acid | < 1.35 g | -0.00037 | < 1.35 g | -0.03555 |
| Choline | < 487.5 mg | -0.00016 |  |  |
| Folate |  |  | > 1000 mg | -0.00002 |
| Niacin | > 35 mg | -0.00090 | > 35 mg | -0.21002 |
| Vitamin A | < 800 µg | -0.00001 | < 800 µg | -0.00010 |
| Vitamin C | < 82.5 mg | -0.00053 | < 82.5 mg | -0.00411 |
| Vitamin D | < 15 µg | -0.02822 | < 15 µg | -0.04681 |
| Vitamin K | < 105 µg | -0.00095 | < 105 µg | -0.00100 |
| Calcium |  |  | < 1000 mg | -0.00002 |
| Iron | > 45 mg | -0.00054 | < 13 mg | -0.99392 |
| Potassium | < 3000 mg | -0.00007 | < 3000 mg | -0.00037 |
| Sodium | < 1500 mg | -0.00010 |  |  |
| Zinc |  |  | < 9.5 mg | -0.64780 |

**Supplementary References**

1. United States Department of Agriculture (USDA), Agricultural Research Service, Nutrient Data Laboratory. USDA National Nutrient Database for Standard Reference, Legacy. Version Current: April 2018. Washington, DC: USDA, 2018. Available from http://www.ars.usda.gov/nutrientdata

2. United States Department of Agriculture (USDA), Agricultural Research service, Nutrient Data Laboratory. USDA National Nutrient Database for Standard Reference, Release 28. Version Current: May 2016. Washington, DC: USDA, 2016. Available from http://www.ars.usda.gov/nea/bhnrc/mafcl

3. Institute of Medicine (IOM). Dietary reference intakes. The essential guide to nutrient requirements. Washington, DC: National Academies Press, 2006. doi: 10.17226/11537

4. Institute of Medicine (IOM). Dietary reference intakes for energy, carbohydrate, fiber, fat, fatty acids, cholesterol, protein, and amino acids. Washington, DC: National Academies Press, 2002/2005. doi: 10.17226/10490

5. National Academies of Sciences, Engineering, and Medicine (NASEM). Dietary reference intakes for energy. Washington, DC: National Academies Press, 2023. doi: 10.17226/26818

6. Institute of Medicine (IOM). Dietary reference intakes for calcium and vitamin D. Washington, DC: National Academies Press, 2011. doi: 10.17226/13050

7. Institute of Medicine (IOM). Dietary reference intakes for vitamin A, vitamin K, arsenic, boron, chromium, copper, iodine, iron, manganese, molybdenum, nickel, silicon, vanadium, and zinc. Washington, DC: National Academies Press, 2001. doi: 10.17226/10026

8. Institute of Medicine (IOM). Dietary reference intakes for vitamin C, vitamin E, selenium, and carotenoids. Washington, DC: National Academies Press, 2000. doi: 10.17226/9810

9. Institute of Medicine (IOM). Dietary reference intakes for thiamin, riboflavin, niacin, vitamin B6, folate, vitamin B12, pantothenic acid, biotin, and choline. Washington, DC: National Academies Press, 1998. doi: 10.17226/6015

10. Institute of Medicine (IOM). Dietary reference intakes for calcium, phosphorus, magnesium, vitamin D and fluoride. Washington, DC: National Academies Press, 1997. doi: 10.17226/5776

11. Institute of Medicine (IOM). Dietary reference intakes for water, potassium, sodium, chloride, and sulfate. Washington, DC: National Academies Press, 2005. doi: 10.17226/10925

12. National Academies of Sciences, Engineering, and Medicine (NASEM). Dietary reference intakes for sodium and potassium. Washington, DC: National Academies Press, 2019. doi: 10.17226/25353
